# A safe, low-cost, easy-to-use 3D camera platform to assess risk of obstructed labor due to cephalopelvic disproportion

**DOI:** 10.1371/journal.pone.0203865

**Published:** 2018-09-14

**Authors:** Rudolph L. Gleason, Mahlet Yigeremu, Tequam Debebe, Sisay Teklu, Daniel Zewdeneh, Michael Weiler, Nate Frank, Lorenzo Tolentino, Shehab Attia, J. Brandon Dixon, Catherine Kwon, Anastassia Pokutta-Paskaleva, Katie A. Gleason

**Affiliations:** 1 The George W. Woodruff School of Mechanical Engineering, Georgia Institute of Technology, Atlanta, GA, United States of America; 2 The Wallace H. Coulter Department of Biomedical Engineering, Georgia Institute of Technology, Atlanta, GA, United States of America; 3 Because of Kennedy, Inc., Acworth, GA, United States of America; 4 Department of Obstetrics & Gynecology, Addis Ababa University, Addis Ababa, Ethiopia; 5 Department of Radiology, Addis Ababa University, Addis Ababa, Ethiopia; 6 LymphaTech, Inc., Atlanta, GA, United States of America; Liverpool John Moores University, UNITED KINGDOM

## Abstract

Cephalopelvic disproportion (CPD)-related obstructed labor is accountable for 3–8% of the maternal deaths worldwide. The consequence of CPD-related obstructive labor in the absence of a Caesarian section (C/S) is often maternal or perinatal mortality or morbidity to the mother and/or the infant. Accurate and timely referral of at-risk mothers to health facilities where C/S is a delivery option could reduce maternal mortality in the developing world. The goal of this work was to develop and test the feasibility of a safe, low-cost, easy-to-use, portable tool, using a Microsoft Kinect 3D camera, to identify women at risk for obstructed labor due to CPD. Magnetic resonance imaging (MRI) scans, 3D camera imaging, anthropometry and clinical pelvimetry were collected and analyzed from women 18–40 years of age, at gestational age ≥36+0 weeks with previous C/S due to CPD (*n* = 43), previous uncomplicated vaginal deliveries (*n* = 96), and no previous obstetric history (*n* = 148) from Addis Ababa, Ethiopia. Novel and published CPD risk scores based on anthropometry, clinical pelvimetry, MRI, and Kinect measurements were compared. Significant differences were observed in most anthropometry, clinical pelvimetry, MRI and Kinect measurements between women delivering via CPD-related C/S versus those delivering vaginally. The area under the receiver-operator curve from novel CPD risk scores base on MRI-, Kinect-, and anthropometric-features outperformed novel CPD risk scores based on clinical pelvimetry and previously published indices for CPD risk calculated from these data; e.g., pelvic inlet area, height, and fetal-pelvic index. This work demonstrates the feasibility of a 3D camera-based platform for assessing CPD risk as a novel, safe, scalable approach to better predict risk of CPD in Ethiopia and warrants the need for further blinded, prospective studies to refine and validate the proposed CPD risk scores, which are required before this method can be applied clinically.

## Introduction

Obstructed labor accounts for 3–8% of all maternal deaths worldwide and, in many countries, is almost as prevalent today as it was 30 years ago [1–3]. Cephalopelvic disproportion (CPD) is an inadequate size of the maternal pelvis, compared to the fetal head, which prevents the fetus from passing through the pelvic cavity during delivery causing obstructed labor [4]. CPD-related obstructed labor requires delivery via Caesarean section (C/S). In the developed world, even if an Obstetrician suspects that a first-time pregnancy is at risk of CPD-related obstructed labor, the clinician will often prescribe trial of labor and, if the labor fails to progress, an emergency C/S is performed. Given the nearly universal access to C/S in much of the developed world, the utility of diagnosing CPD risk has been diminished because C/S capabilities are almost always nearby.

In rural Ethiopia, and many low-resource settings around the world, it may require hours or days of travel for mothers to reach facilities with the infrastructure and expertise to perform a C/S. The consequence of CPD-related obstructive labor (in the absence of C/S) is often maternal and/or perinatal mortality or long-term morbidity to the mother and/or the infant. CPD is especially prevalent in regions like Ethiopia, where girls are small in stature, grow up malnourished, marry at a young age, and become pregnant before the pelvis is fully grown [5, 6]. Maternal and perinatal mortality in Ethiopia are among the highest in the world [7]; up to 22% of maternal deaths in Ethiopia have been attributed to CPD-related obstructed labor [7–9]. Further, maternal morbidity (e.g., obstetric fistulas) from CPD is widespread in Ethiopia and carries significant stigma and social exclusion [10]. In Ethiopia, 1 out of every 250 women report having an obstetric fistula from obstructed labor [11]. Taken together, there is a pressing need to identify safe, low-cost methods for timely and accurate assessment of risk of obstructed labor from CPD in low resource settings [12].

Mengert summarized well that “there are five components of CPD [related obstructed labor]: (1) size and shape of the bony pelvis, (2) size of the fetus, (3) force exerted by the uterus, (4) moldability of the head, and (5) fetal presentation and position” [13]. Over the last century, many have undertaken the aim of developing methods to accurately assess the risk of CPD, prior to labor, based on external and internal physical measurements to assess the size of the pelvis, radiological techniques to assess and compare the pelvic capacity and the fetus size, or anthropometry; the latter is based on the assumption that the size of the pelvis (and perhaps the size of the fetus) correlate, to some degree, with other more clinically tractable measurements of maternal size, such as height or foot size.

Radiological pelvimetry, using X-ray, magnetic resonance imaging (MRI), or computed tomography (CT), can provide accurate quantification of the pelvic planes and fetal size, from which a number of CPD risk scores have been proposed [13–20]; however, the accuracy of radiological pelvimetry at predicting CPD-related obstructive labor is controversial and lacks testing via rigorous randomized trials [14, 21]. Further, MRI and CT are cost prohibitive in low-resource settings and X-ray poses an unwanted health risk from radiation exposure to the fetus and may represent a significant barrier to widespread scale-up in the developing world. Assessment of fetal size is routinely done via ultrasound imaging; however, ultrasound technology and expertise are also limited in most low-resource settings.

Anthropometric features such as maternal height [22–46], foot length [25, 35, 42, 45, 47], shoulder diameter [35], lower limb length [42], BMI [32, 48–51], maternal head circumference-to-height ratio [32], and external measurements to assess hip and pelvis dimensions [28, 34, 35, 45, 52] have been proposed to assess risk of CPD. Often, their diagnostic capabilities rely heavily on the accuracy of the measurements, which contain significant inter- and intra-user variability [53, 54]. CPD risk assessment based on anthropometry have shown low sensitivity and specificity [32–35, 45, 52, 55, 56]. Further, clinical pelvimetry to assess the size of the pelvic cavity by means of the systematic vaginal palpation of specific bony landmarks has shown marginal success and requires skilled personnel and training [57, 58].

With the recent explosion in 3D camera technology, driven in large part by video gaming and virtual reality applications, the resolution, ease-of-use, and widespread availability of 3D cameras, including smartphone-based 3D cameras, are expected to increase dramatically in the years ahead, while cost is expected to continue to fall. The Microsoft Kinect 3D camera, together with innovative software, can generate a 3D point-cloud of the objects in the field of view and uses skeletal mapping algorithms to approximate the location of key joints. Given the required cost, infrastructure, and expertise required for MRI pelvimetry and the inter- and intra-user variability of anthropometry, we hypothesize that a 3D camera-based platform for assessing CPD risk could provide a novel, scalable approach to better predict risk of CPD in the rural developing world. The goal of this paper is to develop and test the feasibility of using a low-cost, easy-to-use, portable technology using a Microsoft Kinect 3D camera to quantify risk of CPD-related obstructed labor.

## Materials and methods

### Participant enrollment and baseline data

This study was approved by the Institutional Review Board Committees at Addis Ababa University, College of Health Sciences (Protocol number: 054/15/gyn, approved 2/5/2016) and the Georgia Institute of Technology (Protocol number: H15314, approved 2/24/16). Women 18 to 40 years of age at gestational age 36 weeks and 0 days (denoted 36+0) or above with vertex presentation and singleton pregnancy that fit into one of the follow groups were recruited between April and November 2016 from Tikur Anbessa (Black Lion) Specialized Referral Hospital in Addis Ababa, Ethiopia: *Group 1*: *High risk*, (*n* = 43): Women with a previous Caesarian section due to CPD and no previous vaginal delivery; *Group 2*: *Low risk* (*n* = 96): Women with at least one previous uncomplicated vaginal delivery and no previous Caesarian sections; *Group 3*: *Unknown risk*, (*n* = 148): Primigravida women. Women in labor, with non-vertex presentation, with Diabetes mellitus, preeclampsia or other hypertensive disease, women that had a previous C/S for reason other than CPD, women that did not intend to try labor (i.e., had a planned C/S), and women with contraindication to MRI were excluded. Written informed consent was obtained from the participant prior to enrollment. Based on both obstetric history and outcome of the current pregnancy, we categorized subjects into two groups: CPD or VD. The CPD group included all women with at least one CPD-related C/S; namely, all women from Group 1 and all women that delivered via CPD-related C/S from Group 2 and Group 3. The VD group included all women that delivered vaginally, with no current or previous C/S; namely, vaginal deliveries from Group 2 and Group 3. We excluded non-CPD-related C/S from Groups 2 and Group 3.

### Participant information

Age, gestational age, systolic and diastolic blood pressure, and weight were recorded from the hospital card of each participant. The number of previous live births and how each previous live birth was delivered (vaginally/instrumentally or C/S) were recorded. For subjects that have had a previous C/S, the Obstetrician and Gynecologist reviewed the hospital card describing the previous deliveries to assess the reason for the previous C/S and, if necessary, confirmed the reason with the participant.

### Anthropometry

Body *height* was measured with a stadiometer (Infant/Child/Adult ShorrBoard®, Weights and Measures, LLC, USA). *Head circumference was* measured as the circumference at the level of the mid-forehead and 1.5 cm above the ear [32]. *Bisacromial (shoulder) width* measured as the distance between the lateral extremities of the acromion processes [35]. *Shoulder height* was measured from the top of the acromion process to the floor. *Waist circumference* was measured at the navel, at the level below the lowest rib, and at the narrowest diameter of the back. *Waist height* was measured as the distance from the naval to the floor. *Hip circumference* was measured at the widest part of the hips and *hip height* was measured from widest part of the hips to the floor. *Foot length* was measured from back of heel to the tip of the big toe [35]. *External conjugate* was measured with an anthropometer (Sammons Preston, Large) as the distance from the point below the lowest lumbar vertebra to the upper edge of the pubic symphysis [45]. All measurements, except for height and external conjugate, were made with a 200 cm non-stretching anthropometry tape measure. All measurements were made by nurses trained by the research team and under the direct observation of the research team. All measurements were recorded to the nearest millimeter. Ratios and differences between the several anthropometric features were calculated.

### Clinical pelvimetry

Clinical pelvimetry was performed by an Obstetrician and Gynecologist (authors MY and ST) following published measurement definitions [58–60]. The *diagonal conjugate* was scored as ‘reachable’ or ‘unreachable’. The *pelvic side walls* were scored as ‘divergent’, ‘straight’, or ‘convergent’. The *sacrospinus ligament* was scored as accommodating two fingers (‘yes’ or ‘no’). The *ischial spines* were scored as ‘prominent’ or ‘not prominent’. The *subpubic angle* was scored as accommodating two fingers (‘yes’ or ‘no’). The *intertuberous ligament* was scored as accommodating the knuckles of a fist (‘yes’ or ‘no’). The *fetal head station* (the position of the fetus' head in relation to the distance from the ischial spines) was scored as -3, -2, -1, 0, 1, 2, or 3 cm from the ischial spine; negative numbers indicate that the head is further inside than the ischial spines and positive numbers show that the head is below the level of the ischial spines.

### Radiological pelvimetry by MRI

Radiological pelvimetry was performed following published methods [18, 61–65]. MRI was performed using a 1.5-Telsa MRI (Achieva Philips) at Tikur Anbessa Specialized Referral Hospital while the patient was in a supine position, at a 30-degree angle, with their head up. A fast-spin echo (FSE) proton density-weighted pulse sequence was used, which is the most commonly used fetal imaging modality of MRI [66]. A T2-Turbo Spin Echo sequence was used with 1200 msec repetition time, 80 msec echo time, 80–102 cm field of view, 276-292-by-316-332 matrix, 1–2 averages, echo number 1, 5 mm section thickness, and 6.0–6.6 mm spacing between slices.

The pelvic inlet (superior strait) is bounded posteriorly by the promontory and alae of the sacrum, laterally by the linea terminalis, and anteriorly by the horizontal rami of the pubic bones and pubis symphysis. The *pelvic inlet anterior-posterior diameter* (*APD*), also called the *obstetric conjugate*, was measured from the anterior cortical surface of the sacrovertebral angel (promontory) to the closest point on the convex posterior-superior aspect of the pubis symphysis. The *diagonal conjugate* was measured from the lower border of the pubic symphysis to the sacral promontory. The *pelvic inlet transverse diameter* (*TD*) was measured as the greatest linear width of the linea terminalis on either side at the level of the sacral promontory. The *pelvic inlet oblique diameters* were measured from one of the sacroiliac synchondroses to the iliopectineal eminence on the opposite side of the pelvis and are designated right and left, according to whether they originate at the right or left sacroiliac synchondrosis.

The midpelvis is at the level of the ischial spines and is the plane of smallest pelvic dimensions. The *mid pelvis APD* was measured from the posterior-inferior aspect of the pubis symphysis to the anterior cortical surface of the sacrococcygeal junction. The *mid pelvis TD* (also called the *interspinous diameter*) was measured as the distance between the inschial spines. The *pelvic outlet APD* was measured from the lower margin of the symphysis pubis to the tip of the sacrum. The *pelvic outlet TD* (also known as the *intertuberous diameter*) was measured between the inner edges of the ischial tuberosities. The *pelvic outlet posterior sagittal diameter* (*PSD*) extends from the tip of the sacrum to a right-angle intersection with a line between the ischial tuberosities. The *vertical Michaelis sacral rhomboid dimension* was measured as the distance between the spinus process tip of the fifth lumbar vertebra to fifth sacral vertebra; the *transverse Michaelis sacral rhomboid dimension* was measured as the distance between the superior posterior iliac spines. The *femoral head separation* was measured as the distance between the medial edge of the right and left femoral heads. The *intertrochanteric distance* was measured as the distance between the lesser trochanter of the left and right femur.

The *fetal head biparietal diameter* (*BPD*) was measured as the transverse diameter on an axial plane that traverses the thalami, and cavum septum pellucidum, which extends from one parietal boss to the other. The *occipitofrontal diameter* (*OFD*) was measured as the anterior-posterior diameter from just above the root of the nose to the most prominent portion of the occipital bone. The *occipitomental diameter* (*OMD*) was measured from the chin to the most prominent portion of the occiput. The *suboccipitobregmatic diameter* (*SOBD*) was measured from the middle of the large fontanel to the undersurface of the occipital bone just where it joins the neck. The *fetal abdomen APD* and *TD* were measured from a transverse section through the upper abdomen that demonstrate the fetal stomach, umbilical vein, and portal sinus.

Pelvimetry and fetal head measurements were obtained by using midsagittal, transverse, and coronal oblique planes with the scan range covering the entire placenta to below lesser trochanters and oblique section through pubic symphysis and sacral promontory. The pelvic inlet, mid-pelvis, and pelvic outlet *APD*, the pelvic *PSD*, and the vertical sacral rhomboid dimension were measured from the midsagittal plane. The pelvic inlet oblique diameters and inter-femoral trochanteric distance were measured in the oblique coronal scan. The pelvic inlet, mid-pelvis, and pelvic outlet *TD*’s, the transverse sacral rhomboid dimension, and the inter-femoral head distance were measured in the transverse plane. The fetal measurements were made on the planes showing the correct reference structures. Fetal head *BPD* and *OFD* were measured in the trans-thalamic axial section of the fetal head. Fetal head *OMD* and *SOBD* were measured in the midsagittal section. Fetal abdomen *APD* and *TD* were measured from the transverse section of the fetal abdomen at the level of the liver where the intrahepatic umbilical vein and stomach is visible. All measurements were made by a trained radiologist. Measurements were taken using RadiAnt DICOM Viewer version 2.2 and recorded to the nearest millimeter.

The pelvic inlet, mid-pelvis, and pelvic outlet circumferences, areas, and pelvic capacities were estimated as *C* = *π*(*TD* + *APD*)/2, *A* = *π*(*TD* + *APD*), and *V* = *πd*^3^/6, respectively, where *d* is the smaller of the *TD* and *APD* in the respective plane. The fetal head *C* and *A* were also quantified at the level of the *BPD* and *OFD* and fetal abdomen *C* and *A* were calculated; the fetal head volume was estimated as *V* = *π*(*BPD*)(*SOBD*)(*OFD* + *OMD*)/12. Ratios between the specific diameters, circumferences, areas, and volumes of the fetus, with those of the pelvic inlet and mid-pelvis were calculated. The fetal-pelvic index (*FPI*) was calculated by adding the two highest circumference differences between the fetal head or abdomen circumference minus the inlet or mid-pelvis circumference [19]. The index of Abitbol *et al*., was calculated as the difference between the fetal *BPD* and the smaller of the inlet *APD* or mid-pelvis *TD* [16]. Following Friedman and Taylor and Sporri *et al*., the difference between the fetal head volume and the inlet, the mid-pelvis, and the smaller of the inlet and mid-pelvis capacity were calculated [17, 18].

### Kinect scanning

**Fig 1:**
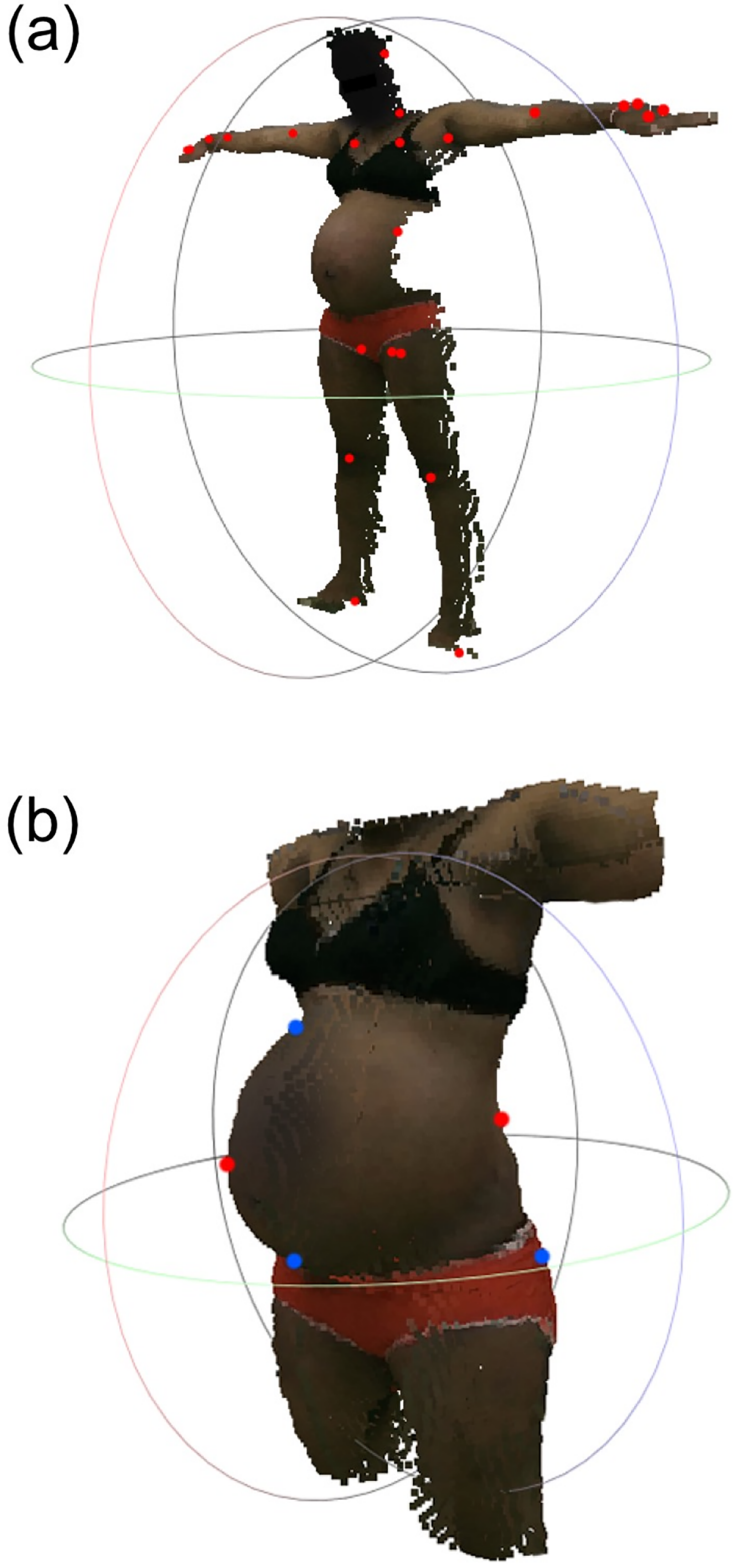
Illustrative kinect image and 3D reconstruction. (a) Illustrative image, point cloud, and skeletal mapping overlay from an anterior view with joint locations from skeletal mapping shown as red dots. Note that the color of the face was darkened to protect the identity of the subject. (b) Illustration of the resulting three-dimensional model of the torso region obtained by stitching together the surface maps from four orthogonal views (anterior, left lateral, posterior, and right lateral). The red and blue dots represent the boundary points that were matched across adjacent scans.

A Kinect v2 sensor (Model #1520, Microsoft, Inc.) with Kinect for Windows SDK 2.0 software was employed to collect clips of the participants in the anterior, posterior, and left and right lateral views, while in a standing position with their legs positioned ~0.5 meters apart and their arms raised at shoulder height in the lateral direction (Fig 1A). The Kinect software algorithms determine the approximate locations of body parts and approximates the location of 24 key joints from the 3D point-cloud. From this skeletal map, custom algorithms to calculate shoulder width, shoulder height, hip width, hip height, hip-to-shoulder length, head height, face height, neck height, shoulder-to-head length, right and left femur length, and right and left leg length from the anterior scan were developed.

Fully-automated algorithms to detection of anthropometric features from anterior and posterior 3D point clouds were developed. Each algorithm employed statistical analysis and gradient analysis on the 3D point cloud data to identify the feature. From the posterior 3D point-clouds, algorithms determined height (distance from the top of the head to the floor), shoulder height (distance from the top of the shoulder directly above the arm pit to the floor), hip height (distance from the widest part of the hip to the floor), torso length (length from v-point at the arm pit to the v-point where the legs meet the torso), hip-to-shoulder length (difference between the shoulder height and hip height), hip diameter (the widest diameter in the pelvic area), waist diameter (the smallest diameter of the torso), shoulder diameter (the distance between the left to right arm pit), and five diameters equally spaced between the hip and shoulders (denoted diameter 1, 2, 3, 4, and 5). From the anterior 3D point-clouds we determined head length (distance from the top of the head to the bottom of the chin), head diameter (widest diameter of the head), fundal height (measured from the pubic symphysis to uppermost point of the fundus), and belly height (the distance of the fundus from the plane made by the pubis and the crest of belly). All landmarks were consistent after manual verification by visually inspection of the point cloud, with the features overlaid on each scan.

In addition to assessing measurements from point-clouds from individual scans, algorithms were developed to ‘stitch’ the point-clouds of the torso together from the four orthogonal views into an accurate 3D model of that participant (Fig 1), using an iterative closest point (ICP) algorithm. The ICP algorithm is well-studied and commonly used in image processing to match features between adjacent frames in two or more images to stitch them together. The algorithm relies on two main features to successfully stitch two images, or in our case, point clouds: (*i*) initial transformations of the point clouds that will be stitched; and, (*ii*) matching surface features or reference points on the adjacent point clouds. An initial transformation was achieved by translating and rotating the left, right lateral scans and posterior scans to be in the correct anatomical direction, relative to the anterior scan. Challenges arose in matching surface features and creating reference points using scans that are adjacently orthogonal due to the lack of similar features between adjacent scans; e.g., the anterior torso scan may have a curved belly and a symmetric shape; however, the left torso lateral scan lacks the symmetry of its partner and the Kinect may not capture the full belly from the sides, hence there would not be an adequate number of references for the ICP algorithm to match the two scans. To overcome this issue, we used the geometrical shape of the edges of the scans in 2D and then 3D as reference points. The edges, such as the curvature of the anterior scan from the shoulder, along the hips and down to the thigh, matched the curvature of the left and right lateral scans along similar sides, providing adequate information for the ICP algorithm to reconstruct the shape of the subject.

### Pregnancy outcomes

After delivery, maternal and perinatal mortality, birth date, method of delivery, reason for C/S, and baby weight, length, head circumference, and Apgar score were recorded. The pregnancy outcome was recorded as “Vaginal Delivery (VD)” or “Caesarean Section (C/S)” by review of the hospital card after delivery. For delivery via C/S, the hospital card was reviewed and, if necessary discussion with the attending physician was conducted, to assess the reason that C/S was prescribed. Reason for C/S were recorded in the following categories: CPD after prolonged labor, elective C/S due to previous CPD (Group 1 only), abnormal presentation, fetal distress, placental concerns, failed induction, or other.

### Risk score framework

**Fig 2:**
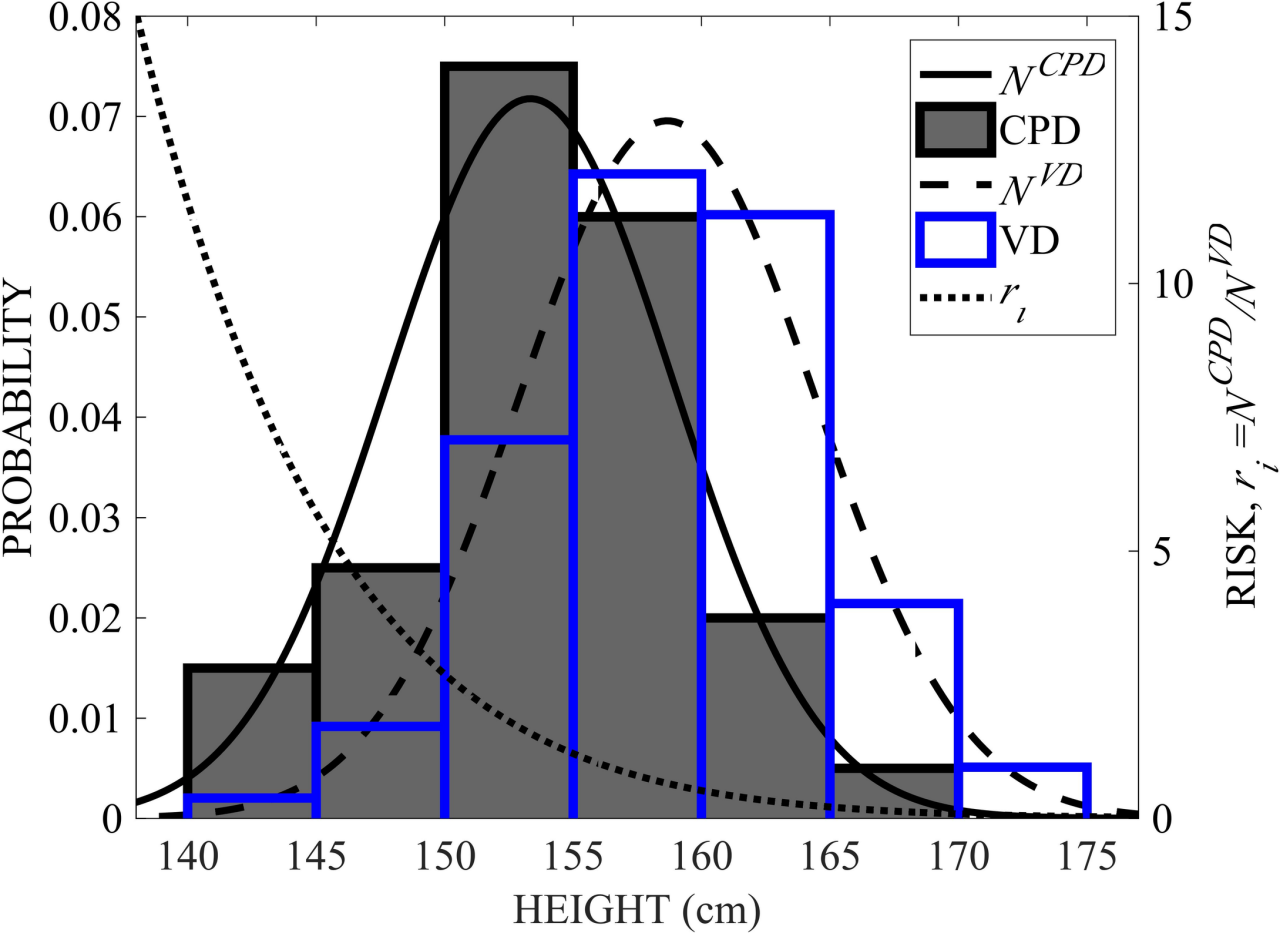
Illustrative risk model, *r_i_*. Histograms and Gaussian distribution functions of feature fi = height for all CPD cases (shaded histogram bars and solid line NCPD) and all uncomplicated vaginal delivery cases (blue histogram bars and dashed line NVD) and $r_{i}=(N_{i}^{CPD}/N_{i}^{VD})$ from Eq (2)_2_ is shown in the dotted line. Note that the histograms and distribution functions refer to the vertical axis to the left and ri refers to the vertical axis to the right.

We performed binomial logistic regression to develop predictive risk scores based on either (*i*) anthropometry, (*ii*) clinical pelvimetry, (iii) MRI, or (iv) Kinect measurements. The risk score was defined as

$$R^{j}=\beta_{o}+\sum_{i=1}^{N}\beta_{i}r_{i}$$ (1)

where *j* = *A*,*P*,*K*,*M* denotes risk scores based on anthropometry, clinical pelvimetry, Kinect 3D camera, or MRI measurements, respectively, and *β*_*o*_ and *β*_*i*_ are model parameters. We considered two different models for *r*_*i*_; namely,

$$r_{i}=x_{i}\;or\;r_{i}=\frac{N_{i}^{CPD}(x;\mu_{i}^{CPD},\sigma_{i}^{CPD})}{N_{i}^{VD}(x;\mu_{i}^{VD},\sigma_{i}^{VD})}$$ (2)

where *x*_*i*_ are the values of the features *f*_*i*_ (e.g., *f*_*i*_ = height, *x*_*i*_ = 155 cm) measured with the respective tool, $N_{i}^{CPD}(x;\mu_{i}^{CPD},\sigma_{i}^{CPD})$ and $N_{i}^{VD}(x;\mu_{i}^{VD},\sigma_{i}^{VD})$ are the normal Gaussian probability distribution functions of the feature *f*_*i*_ for women who delivered via CPD-related C/S or uncomplicated vaginal delivery, respectively, $\mu_{i}^{CPD}$ and $\mu_{i}^{VD}$ are means and $\sigma_{i}^{VD}$ and $\sigma_{i}^{CPD}$ standard deviations for women who delivered via CPD-related C/S or via vaginal delivery, respectively. To illustrate, $N_{i}^{CPD}(x_{i};\mu_{i}^{CPD},\sigma_{i}^{CPD})$, $N_{i}^{VD}(x_{i};\mu_{i}^{VD},\sigma_{i}^{VD})$, and *r*_*i*_ are shown for *f*_*i*_ = *height* (Fig 2). We denote models using Eq (2)_1_ as r_1_ and those using Eq (2)_2_ as r_2_. Since the independent variables for clinical pelvimetry are categorical, r_2_ was not considered for clinical pelvimetry models.

### Risk score assessment

Best fit models were determined using the glmfit subroutine in MATLAB (MathWorks) for $n_{p}=\frac{l_{j}!}{k!(l_{j}-k)!}$ possible permutations of feature sets and parameters were calculated for each value of *k* for all subjects, where $l_{j}$ is the total number of features considered for inclusion in the model and *k* is the actual number of features included in the model. For each of the *n*_*p*_ parameter sets, for each value of *k*, the area under the ROC curve, denoted *AUC*, was calculated with the perfcurve MATLAB subroutine and the best model was defined as the feature set and model parameters with the greatest *AUC*, denoted *AUC*_*best*_. For the pelvimetry- and anthropometry-based models, the total number of features was $l_{J}$ = 7 and 21, respectively; for these models, we calculated the best fit parameters (*β*_*o*_ and *β*_*i*_) for all *n*_*p*_ possible permutations of feature sets.

For Kinect and MRI models, $l_{J}$ = 54 and 49, respectively; for these models, *n*_*p*_ was far too large to compute all possible permutations of parameter sets. Therefore, we devised a parameter elimination routine for cases when $\frac{l_{j}!}{k!(l_{j}-k)!}$ was above 30-million permutations. Features were eliminated based on the percent of occurrences that each feature appears in the top 1,000 models from the models with lower feature counts *k* (forward elimination) and independently from models with a higher feature count (backward elimination); parameters with the lowest occurrences in models with lower (or higher) feature count were eliminated, thereby reducing $l_{j}$ for that parameter count *k* and yielding a manageable *n*_*p*_ that was <10-million. Based on the forward and backward elimination routines, all evaluated models were ranked by *AUC* and the percentage of time that each feature appears in the top 10,000 models was calculated. Another ~10-million permutations were evaluated, wherein the features that appeared most often in the top models were fixed (i.e., not permutated) and the features that appeared less often were permutated. The models were again ranked by *AUC* and *AUC*_*best*_ was the feature set and parameters associated with the model with the highest *AUC* from all permutations considered.

Since all of the data were used to identify the best model, these models were over-fitted (i.e., optimistic). To evaluate the optimism, *O*, and to assess the predictive capabilities of the best models, we adopted the approach of Harrell et al. [67]. Briefly, *n*_*B*_ = 2,000 non-parametric bootstrap samples were generated from the original data set. For each bootstrap sample set, the top feature sets were selected and the model parameters (*β*_*o*,*b*_ and *β*_*i*,*b*_) were identified and the *AUC* for each bootstrap sample, denoted *AUC*_*b*,*boot*_, was calculated. The feature set and fitted model parameters (*β*_*o*,*b*_ and *β*_*i*,*b*_) were then applied to the original data set and the *AUC*, denoted *AUC*_*b*,*orig*_, was calculated. The optimism was calculated as

$$O=\frac{1}{n_{B}}\sum_{b=1}^{n_{B}}\left({AUC}_{b,boot}-{AUC}_{b,orig}\right)$$ (3)

The adjusted *AUC*, denoted *AUC*_*adj*_, which represents the expected *AUC* if the model was applied to a new data set (i.e., the predictive capability), was calculated as

$${AUC}_{adj}={AUC}_{best}-O.$$ (4)

For each number of features, *k*, *O* and *AUC*_*adj*_ were calculated for the top 200 feature sets and parameters with the highest *AUC*_*best*_. The most predictive model was determined as the model with the highest *AUC*_*adj*_.

A 2x2 contingency table and 4-point risk score (*very high*, *high*, *moderate*, and *mild risk*) table were determined for the model with the highest *AUC*_*adj*_. The cut-off risk score for the 2x2 contingency table and for the *high*-*moderate* cut-off was set as the value in which the true positive rate (*TPR*, a.k.a., *sensitivity*) was equal to the true negative rate (*TNR*, a.k.a., the *specificity*). The *very high-high* cut-off was the highest risk value where the false positive rate (*FPR*) was ≤10%. We define the *detection rate* as the percentage of CPD cases that score as very high risk. The *moderate-mild* cut-off was the highest risk value where the false negative rate (*FNR*) was ≤10%. The likelihood ratios for CPD and VD are defined as ${LR}_{CPD}=\frac{TPR}{FPR}$ and ${LR}_{VD}=\frac{TNR}{FNR}.$

### Sample size determination and statistical analysis

Given that use of the Kinect 3D camera is a new strategy to predict CPD risk there are no data in the literature that can accurately estimate expected standard deviations or correlations between experimental Kinect-based measures across groups. Indeed, the purpose of this study was to generate the first data on these measures to assess the feasibility of the Kinect-based approach, compared to MRI, anthropometry, and clinical pelvimetry. The sample sizes for each group were selected to (*i*) recruit 50 women considered as high risk for CPD and (*ii*) to quantify the prevalence of CPD in this Ethiopian population in women with no history of CPD and in women with no obstetric history; the latter will inform future sample size determination. The target sample size of 50 cases was based on previous studies in literature, aimed at assessing risk of CPD based on anthropometry [1–6]. We considered all women with a history of CPD as high-risk cases. We estimated the prevalence of CPD in primigravida women to be 3–5%; thus, a sample size of *n* = 148 was expected to yield between 4 and 7 CPD cases. We estimated the prevalence in women with previous uncomplicated vaginal deliveries to be 2–4%; thus, a sample size of *n* = 96 was expected to yield between 2 and 4 CPD cases. Together, these sample sizes were expected to yield 50 high-risk subjects and should yield an adequate estimate of the prevalence of CPD in primigravida and multigravida women with no history of CPD.

For continuous variables, a two-sample *t*-test was performed and for categorical variables, *χ*^2^ analysis was performed to determine statistical significance across groups (*p* < 0.05); for categorical variables with more than two categories, a pairwise Marascuillo procedure was performed to compare differences in proportions across groups for each category (*p* < 0.05). The standard error and 95% confidence interval with the nonparametric assumption was determined for the *AUC* of the ROC curves and statistical significance (*p*<0.05) against the null hypothesis that the *AUC* = 0.50 was reported. Statistical analysis was performed in MATLAB 2018a (MathWorks) and IBM^®^ SPSS^®^ Statistics, version 24.

## Results

### Pregnancy outcomes

**Fig 3:**
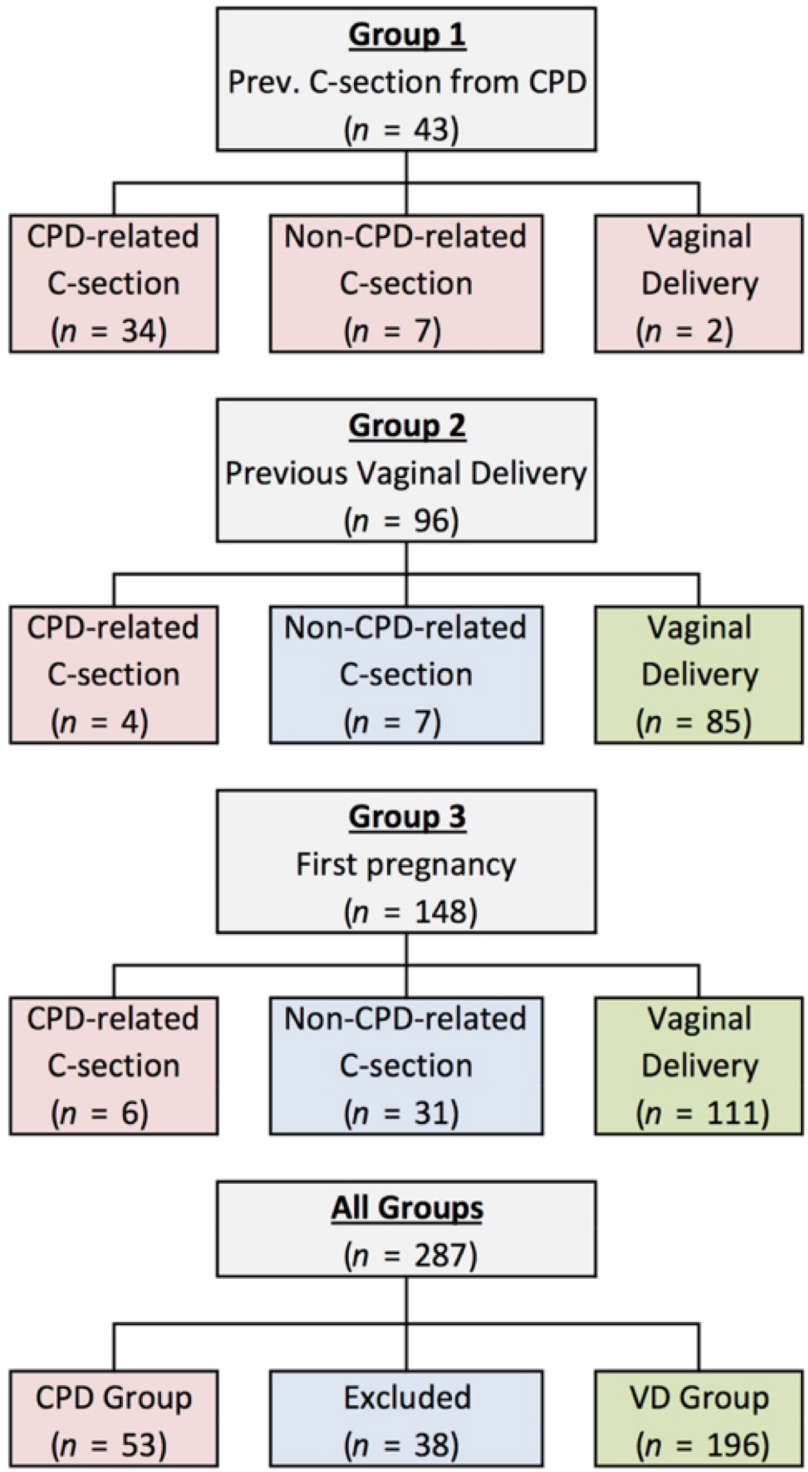
Pregnancy outcomes. Pregnancy outcomes were categorized as CPD-related C/S, non-CPD-related C/S, or VD. Based on both obstetric history and outcome of the current pregnancy, subjects were categorized into two groups: CPD (shaded red) or VD (shaded green). Exclusions are shaded in blue.

The incidence of CPD related C/S was 79%, 4%, and 4%, among women with previous CPD-related C/S, women with a successful previous vaginal delivery, and primigravida women, respectively (Fig 3). Of the 45 non-CPD related C/S (across Groups 1, 2, and 3), 21 were due to fetal distress, 8 were due to failed induction, 3 were due to abnormal presentation, 1 was due to placental issues, and the remaining 12 were due to other reasons. All women in Group 2 and Group 3 tried labor prior to C/S; however, some women in Group 1 underwent a scheduled C/S, without trial of labor. No maternal mortality was observed across all 287 subjects. Perinatal mortality occurred in three deliveries, all following vaginal deliveries in primigravida women (Group 3).

### Measurement differences across CPD and vaginal delivery outcomes

Participant age and 1-minute APGAR scores were higher and parity lower in the CPD group compared to the VD group (Table 1). Total height, shoulder height, waist height, hip height, shoulder width, and foot length were significantly lower and head-circumference-to-height ratio and BMI were significantly higher in the CPD group compared to the VD group. Inlet *APD*, *TD*, right and left *OD*, circumference, cross-sectional area, and capacity, diagonal conjugate, midpelvic *APD*, circumference, and cross-sectional area, and outlet *APD*, circumference, cross-sectional area, and capacity were all significantly smaller in the CPD group compared to the VD group. No differences were observed in any fetal head or fetal abdomen measurements. *BPD* to smallest inlet diameter ratio, fetal head circumference- and fetal abdomen circumference to inlet circumference and to mid-pelvis circumference ratios, and fetal head- and fetal abdomen- to inlet area and mid-pelvis area ratios were all higher in the CPD compared to the VD groups. The indices proposed by Friedman and Taylor (on the pelvic inlet), Sporri *et al*., and *FPI* were significantly different in the CPD group compared to the VD group. The vertical and transverse sacral rhomboid dimension and the distance between the right and left femoral heads was significantly lower in the CPD group compared to the VD group.

**Table 1:** Anthropometry, clinical pelvimetry, MRI, and kinect measurements and novel risk scores. Model parameters (*β*_*o*_ and *β*_*i*_) are for the model specified in Eq (1), with either Eq (2)_1_ or (2)_2_. Eq (2)_1_ was used for the clinical pelvimetry scores since these data were categorical. Eq (2)_1_ outperformed Eq (2)_2_ for the anthropometry risk score, whereas Eq (2)_2_ outperformed Eq (2)_1_ for the MRI and Kinect risk scores. The red bold-italic font denotes features that are statistically different (*p*<0.05) between the CPD and VD groups. Note: CPD = cephalopelvic disproportion, VD = vaginal delivery, cm = centimeters, kg = kilograms, BMI = body mass index, *APD* = anterior-posterior diameter, *TD* = transverse diameter, *AC* = abdomen circumference, *OD* = oblique diameter, *PSD* = posterior sagittal diameter, *BPD* = biparental diameter, *OFD* = occipitofrontal diameter, *OMD* = occipitomental diameter, *SOBD* = suboccipitobregmatic diameter, *HC* = head circumference, and *AC* = abdomen circumference.

			CPD			VD			*p*	*β*
			(*n* = 53)			(*n* = 196)				
***Maternal and Fetal Characteristics ***										
		***Age***	***28*.*1***	***±***	***3*.*9***	***26*.*2***	***±***	***5*.*0***	***0*.*01***	
		Gestational Age at delivery (weeks)	40.7	±	1.2	40.8	±	1.3	0.62	
		***Parity***	***2*.*3***	***±***	***0*.*7***	***2*.*7***	***±***	***0*.*9***	***0*.*01***	
		Newborn Weight (kg)	3.3	±	0.5	3.3	±	0.5	0.67	
		Newborn Length (cm)	52.4	±	3.3	51.6	±	2.9	0.27	
		Newborn Head Circumference (cm)	35.9	±	1.6	35.8	±	1.5	0.81	
		***Apgar score (1 min)***	***7*.*6***	***±***	***0*.*8***	***7*.*9***	***±***	***0*.*5***	***0*.*01***	
		Apgar score (5 min)	8.8	±	0.7	8.9	±	0.5	0.09	
**Anthropometry**										*β*_*o*_ = 15.46
	***Heights***									
		***Total Height (cm)***	***153*.*5***	***±***	***5*.*4***	***158*.*7***	***±***	***5*.*7***	***<0*.*001***	--
		***Shoulder Height (cm)***	***129*.*3***	***±***	***7*.*6***	***132*.*3***	***±***	***6*.*7***	***0*.*006***	--
		***Waist Height (cm)***	***94*.*2***	***±***	***4*.*7***	***97*.*7***	***±***	***4*.*4***	***<0*.*001***	--
		***Hip Height (cm)***	***86*.*7***	***±***	***4*.*6***	***90*.*5***	***±***	***5*.*6***	***<0*.*001***	-0.324
		Hip-to-shoulder length (cm)	42.7	±	6.9	41.8	±	6.4	0.37	--
		Weight (kg)	66.7	±	12.1	66.7	±	9.9	0.99	0.088
	***Circumferences/widths***									
		Head Circumference (cm)	56.5	±	2.2	56.3	±	2.5	0.67	--
		***Shoulder Width (cm)***	***38*.*0***	***±***	***2*.*6***	***39*.*6***	***±***	***2*.*7***	***<0*.*001***	-0.219
		Waist Circumference (cm)	102.2	±	8.2	102.1	±	8.8	0.92	--
		Hip Circumference (cm)	98.5	±	9.6	98.7	±	6.9	0.85	--
		***Foot Length (cm)***	***23*.*7***	***±***	***1*.*1***	***24*.*1***	***±***	***1*.*2***	***0*.*028***	--
		External Conjugate (cm)	21.0	±	2.2	20.9	±	2.2	0.75	--
	***Ratios***									
		***BMI***	***28*.*3***	***±***	***4*.*7***	***26*.*5***	***±***	***3*.*4***	***0*.*002***	--
		Waist-to-hip circumference ratio	1.04	±	0.07	1.04	±	0.07	0.57	--
		***Head circ*. *to height ratio***	***0*.*368***	***±***	***0*.*016***	***0*.*356***	***±***	***0*.*019***	***<0*.*001***	56.014
		***Hip circumference-to-Hip height***	***1*.*14***	***±***	***0*.*11***	***1*.*09***	***±***	***0*.*11***	***0*.*01***	-25.178
		***Shoulder width-to-Hip circumference***	***0*.*39***	***±***	***0*.*03***	***0*.*40***	***±***	***0*.*03***	***0*.*003***	--
		Head circumference-to-Hip circ.	0.58	±	0.05	0.57	±	0.04	0.46	19.281
		Hip height-to-Height	0.56	±	0.03	0.57	±	0.03	0.18	--
		(Hip-to-shoulder length)-to-Hip height	0.49	±	0.09	0.47	±	0.10	0.07	5.941
		***Head circ*.*-to-foot length***	***2*.*39***	***±***	***0*.*13***	***2*.*34***	***±***	***0*.*14***	***0*.*03***	-2.829
**Pelvimetry from Physical Exam**										*β*_*o*_ = 3.73
	***Diagonal conjugate***									-2.231
		***Reached***	***7***	***(13%)***		***2***	***(1%)***		***<0*.*05***	
		***Unreached***	***45***	***(87%)***		***176***	***(99%)***		***<0*.*05***	
	***Pelvic Side Walls***									--
		***Divergent***	***9***	***(17%)***		***65***	***(37%)***		***<0*.*05***	
		***Straight***	***27***	***(52%)***		***101***	***(57%)***		***<0*.*05***	
		***Convergent***	***16***	***(31%)***		***12***	***(7%)***		***<0*.*05***	
	***Sacrospinus ligament accommodates more than 2 fingers*?* ***									--
		***Yes***	***38***	***(73%)***		***173***	***(97%)***		***<0*.*05***	
		***No***	***13***	***(25%)***		***5***	***(3%)***		***<0*.*05***	
	***Are Ischial spines prominent*?**									-1.441
		***Yes***	***18***	***(35%)***		***11***	***(6%)***		***<0*.*05***	
		***No***	***34***	***(65%)***		***167***	***(94%)***		***<0*.*05***	
	***Subpubic angle accommodates more than 2 fingers*?* ***									--
		***Yes***	***43***	***(83%)***		***175***	***(98%)***		***<0*.*05***	
		***No***	***9***	***(17%)***		***3***	***(2%)***		***<0*.*05***	
	***Intertuberous ligament accommodates the knuckles of a fist*?**									1.273
		***Yes***	***39***	***(75%)***		***170***	***(96%)***		***<0*.*05***	
*** ***	*** ***	***No***	***13***	***(25%)***		***8***	***(4%)***		***<0*.*05***	
	***Fetal head station***									-0.295
		*Score = -3*	32	(62%)		68	(38%)		>0.05	
		*Score = -2*	14	(27%)		57	(32%)		>0.05	
		*Score = -1*	4	(8%)		35	(20%)		>0.05	
		*Score = 0*	1	(2%)		16	(9%)		>0.05	
		*Score = 1*	1	(2%)		2	(1%)		>0.05	
		*Score = 2*	0	(0%)		0	(0%)		>0.05	
	*** ***	*Score = 3*	0	(0%)		0	(0%)		>0.05	
**MRI Pelvimetry**										*β*_*o*_ = -28.0
	***Pelvic inlet***									
		***Inlet APD (cm)***	***10*.*3***	***±***	***1*.*0***	***11*.*2***	***±***	***1*.*0***	***<0*.*001***	--
		***Diagonal Conjugate (cm)***	***11*.*6***	***±***	***1*.*0***	***12*.*6***	***±***	***1*.*0***	***<0*.*001***	--
		***Inlet TD (cm)***	***12*.*3***	***±***	***0*.*8***	***12*.*8***	***±***	***0*.*8***	***0*.*001***	0.951
		***Inlet Right OD (cm)***	***12*.*0***	***±***	***0*.*7***	***12*.*5***	***±***	***0*.*8***	***<0*.*001***	--
		***Inlet Left OD (cm)***	***11*.*9***	***±***	***0*.*8***	***12*.*5***	***±***	***0*.*8***	***<0*.*001***	0.522
		***Inlet circumference (cm)***	***35*.*5***	***±***	***2*.*1***	***37*.*7***	***±***	***2*.*2***	***<0*.*001***	--
		***Inlet area (cm***^***2***^***)***	***399***	***±***	***49***	***450***	***±***	***53***	***<0*.*001***	--
		***Inlet capacity (cm***^***3***^***)***	***580***	***±***	***160***	***740***	***±***	***187***	***<0*.*001***	1.568
	***Midpelvis***									
		***Mid-pelvis APD (cm)***	***10*.*7***	***±***	***0*.*8***	***11*.*1***	***±***	***0*.*9***	***0*.*003***	8.733
		Mid-pelvis *TD* (cm)	9.3	±	0.8	9.5	±	0.8	0.080	18.959
		***Mid-pelvis circumference (cm)***	***31*.*4***	***±***	***2*.*0***	***32*.*4***	***±***	***2*.*1***	***0*.*002***	-10.483
		***Mid-pelvis area (cm***^***2***^***)***	***312***	***±***	***40***	***332***	***±***	***44***	***0*.*003***	--
		Mid-pelvis capacity (cm^3^)	428	±	110	458	±	120	0.099	--
	***Pelvic Outlet***									
		***Outlet APD (cm)***	***9*.*9***	***±***	***0*.*8***	***10*.*2***	***±***	***0*.*9***	***0*.*047***	-2.940
		Outlet *TD* (cm)	10.7	±	1.0	11.0	±	0.8	0.051	--
		Outlet *PSD* (cm)	4.9	±	0.8	5.0	±	0.9	0.25	--
		***Outlet circumference (cm)***	***32*.*4***	***±***	***2*.*2***	***33*.*2***	***±***	***2*.*0***	***0*.*009***	16.687
		***Outlet area (cm***^***2***^***)***	***334***	***±***	***46***	***351***	***±***	***42***	***0*.*011***	-19.044
		***Outlet capacity (cm***^***3***^***)***	***494***	***±***	***110***	***530***	***±***	***114***	***0*.*045***	--
	***Fetal Measurements***									
		Fetal head *BPD* (cm)	9.8	±	0.5	9.9	±	0.5	0.41	--
		Fetal head *OFD* (cm)	11.8	±	0.7	11.8	±	0.6	0.46	--
		Fetal head *OMD* (cm)	13.2	±	0.6	13.0	±	0.8	0.17	6.623
		Fetal head *SOBD* (cm)	9.7	±	0.6	9.6	±	0.7	0.81	--
		Fetal *HC* (cm)	34.0	±	1.7	34.0	±	1.6	0.91	131.57
		Fetal Head Area (cm^2^)	366	±	35	366	±	34	0.97	-146.83
		Fetal head volume (cm3)	625	±	87	620	±	83	0.70	19.570
		Fetal abdomen *APD* (cm)	10.6	±	0.9	10.6	±	0.9	0.99	80.082
		Fetal abdomen *TD* (cm)	11.1	±	1.0	11.1	±	0.9	0.92	7.802
		Fetal *AC* (cm)	34.1	±	2.5	34.1	±	2.3	0.95	40.817
		Fetal Abdomen Area (cm^2^)	371	±	53	370	±	51	0.94	-121.77
	***MRI Anthropometry***									
		***Vertical Sacral Rhomboid Dimension***	***10*.*8***	***±***	***0*.*9***	***11*.*1***	***±***	***0*.*9***	***0*.*048***	1.735
		***Transverse Sacral Rhomboid Dimension***	***9*.*1***	***±***	***1*.*2***	***9*.*5***	***±***	***1*.*2***	***0*.*036***	--
		***Femoral head separation (cm)***	***11*.*6***	***±***	***0*.*7***	***12*.*0***	***±***	***0*.*9***	***<0*.*001***	1.479
		Intertrochanteric distance (cm)	17.5	±	1.1	17.8	±	1.4	0.16	--
	***Ratios***									
		***BPD to smallest inlet diameter***	***0*.*97***	***±***	***0*.*10***	***0*.*89***	***±***	***0*.*08***	***<0*.*001***	--
		***Fetal HC-to-Inlet Circumference***	***0*.*96***	***±***	***0*.*08***	***0*.*91***	***±***	***0*.*06***	***<0*.*001***	-3.569
		***Fetal AC-to-Inlet Circumference***	***0*.*96***	***±***	***0*.*08***	***0*.*91***	***±***	***0*.*07***	***<0*.*001***	--
		***Fetal Head Area-to-Inlet Area***	***0*.*93***	***±***	***0*.*16***	***0*.*82***	***±***	***0*.*12***	***<0*.*001***	1.803
		***Fetal Abdomen Area-to-Inlet Area***	***0*.*94***	***±***	***0*.*16***	***0*.*83***	***±***	***0*.*13***	***<0*.*001***	--
		BPD-to-smallest mid-pelvis diameter	1.07	±	0.11	1.05	±	0.10	0.26	--
		***Fetal HC-to-Mid-pelvis Circumference***	***1*.*09***	***±***	***0*.*08***	***1*.*05***	***±***	***0*.*08***	***0*.*009***	--
		***Fetal AC-to-Mid-pelvis Circumference***	***1*.*09***	***±***	***0*.*10***	***1*.*06***	***±***	***0*.*09***	***0*.*017***	--
		***Fetal Head Area-to-Mid-pelvis Area***	***1*.*19***	***±***	***0*.*18***	***1*.*12***	***±***	***0*.*18***	***0*.*012***	-5.132
		***Fetal Abdomen Area-to-Mid-pelvis Area***	***1*.*21***	***±***	***0*.*23***	***1*.*13***	***±***	***0*.*19***	***0*.*015***	--
		Abitbol *et al*.^6^	-0.62	±	0.93	-0.40	±	0.96	0.14	--
		***Friedman & Taylor (Inlet)***^7^	***45***	***±***	***189***	***-119***	***±***	***195***	***<0*.*001***	--
		Friedman & Taylor (Midpelvis)^7^	197	±	145	162	±	155	0.14	--
		Sporri et al.^8^	208	±	144	167	±	151	0.08	-11.803
*** ***	*** ***	***Fetal-pelvic Index (FPI)***^9^	***5*.*5***	***±***	***5*.*2***	***3*.*4***	***±***	***5*.*0***	***0*.*009***	7.026
**Kinects Measurements**										*β*_*o*_ = -4.08
	***Skeletal Mapping Measurements (front)***		* *							
		***Head Height (cm)***	***147*.*1***	***±***	***6*.*2***	***152*.*9***	***±***	***6*.*4***	***<0*.*001***	*--*
		***Face Height (cm)***	***134*.*0***	***±***	***5*.*8***	***139*.*3***	***±***	***5*.*9***	***<0*.*001***	*--*
		***Neck Height (cm)***	***127*.*3***	***±***	***5*.*6***	***132*.*6***	***±***	***5*.*7***	***<0*.*001***	*--*
		***Shoulder Height (cm)***	***126*.*6***	***±***	***5*.*7***	***131*.*8***	***±***	***5*.*9***	***<0*.*001***	*--*
		***Hip Height (cm)***	***79*.*2***	***±***	***4*.*6***	***83*.*4***	***±***	***5*.*0***	***<0*.*001***	*--*
		***Right Leg Length (cm)***	***72*.*3***	***±***	***5*.*4***	***76*.*3***	***±***	***5*.*8***	***<0*.*001***	*--*
		***Left Leg Length (cm)***	***72*.*2***	***±***	***5*.*1***	***76*.*4***	***±***	***5*.*6***	***<0*.*001***	*--*
		***Right Femur Length (cm)***	***29*.*8***	***±***	***3*.*3***	***31*.*3***	***±***	***3*.*3***	***0*.*010***	*--*
		***Left Femur Length (cm)***	***29*.*5***	***±***	***3*.*1***	***30*.*6***	***±***	***3*.*5***	***0*.*045***	1.109
		Hip-to-shoulder length (cm)	47.5	±	3.5	48.4	±	2.7	0.06	--
		***Shoulder-to-head length (cm)***	***20*.*5***	***±***	***1*.*4***	***21*.*1***	***±***	***2*.*0***	***0*.*031***	1.545
		Shoulder Width (cm)	28.3	±	2.8	28.8	±	2.3	0.22	--
		Hip Width (cm)	12.7	±	1.7	12.9	±	1.3	0.35	--
	***3D Camera Measurements***									
		***Height (cm)***	***156*.*2***	***±***	***6*.*3***	***161*.*8***	***±***	***7*.*2***	***<0*.*001***	*--*
		***Shoulder Height (cm)***	***121*.*7***	***±***	***5*.*8***	***126*.*2***	***±***	***5*.*8***	***<0*.*001***	*--*
		***Hip Height (cm)***	***71*.*3***	***±***	***4*.*6***	***74*.*0***	***±***	***8*.*9***	***0*.*045***	*--*
		***Head length (cm)***	***21*.*9***	***±***	***1*.*1***	***22*.*6***	***±***	***1*.*7***	***0*.*012***	*--*
		Torso Length (cm)	51.8	±	3.5	52.8	±	3.0	0.06	1.543
		***Hip-to-shoulder length (cm)***	***48*.*1***	***±***	***3*.*0***	***49*.*4***	***±***	***3*.*8***	***0*.*030***	*--*
		***Hip Dimeter (cm)***	***36*.*2***	***±***	***3*.*3***	***37*.*2***	***±***	***2*.*6***	***0*.*035***	*--*
		***Diameter1 (cm)***	***36*.*0***	***±***	***3*.*1***	***37*.*0***	***±***	***2*.*6***	***0*.*030***	*--*
		Diameter2 (cm)	31.8	±	4.3	32.5	±	3.0	0.18	0.400
		Diameter3 (cm)	28.5	±	3.6	28.7	±	3.1	0.70	2.056
		Diameter4 (cm)	26.8	±	2.3	27.0	±	2.1	0.44	3.719
		Diameter5 (cm)	30.5	±	2.8	31.1	±	2.4	0.09	--
		Shoulder Diameter (cm)	30.7	±	2.8	31.2	±	2.4	0.21	--
		Waist Diameter (cm)	25.4	±	2.2	25.5	±	2.1	0.72	-11.870
		***Head Diameter (cm)***	***12*.*5***	***±***	***1*.*5***	***13*.*3***	***±***	***2*.*1***	***0*.*015***	0.887
		Fundal Height (cm)	44.5	±	8.2	44.7	±	8.3	0.85	--
		Belly Height (cm)	25.2	±	3.6	24.4	±	3.2	0.18	--
	***Ratios***									
		Hip height-to-Hip Diameter (3D)	1.98	±	0.19	2.03	±	0.18	0.09	-2.998
		Head height-to-Hip Width (SM)	11.8	±	1.5	12.0	±	1.2	0.33	--
		***Belly Height-to-Hip Diameter (3D)***	***0*.*70***	***±***	***0*.*11***	***0*.*66***	***±***	***0*.*09***	***0*.*017***	--
		Belly Height (3D)-to-Hip Width (SM)	2.02	±	0.43	1.92	±	0.33	0.06	--
		Fundal Height-to-Hip Diameter (3D)	1.23	±	0.25	1.20	±	0.23	0.39	--
		Fundal Height (3D)-to-Hip Width (SM)	3.57	±	0.81	3.52	±	0.79	0.67	--
		Hip Height-to-(Hip-to-shoulder length) (SM)	1.71	±	0.15	1.75	±	0.16	0.13	-7.328
		Head Height-to-(Hip-to-shoulder length) SM	3.17	±	0.16	3.22	±	0.19	0.08	5.270
		Shoulder diameter-to-Hip diameter (3D)	0.85	±	0.06	0.84	±	0.06	0.71	--
		Shoulder width-to-Hip width (SM)	2.26	±	0.27	2.25	±	0.18	0.74	0.018
		Height-to-Hip height (3D)	2.19	±	0.08	2.17	±	0.11	0.18	2.804
		***Head height-to-hip height (SM)***	***1*.*86***	***±***	***0*.*07***	***1*.*84***	***±***	***0*.*06***	***0*.*012***	0.841
		***Torso length-to-Hip height (3D)***	***0*.*73***	***±***	***0*.*06***	***0*.*70***	***±***	***0*.*05***	***0*.*005***	0.818
		***Height-to-Belly height (3D)***	***6*.*34***	***±***	***0*.*97***	***6*.*72***	***±***	***0*.*84***	***0*.*007***	--
		***Head height (SM)-to-Belly height (3D)***	***5*.*97***	***±***	***0*.*91***	***6*.*35***	***±***	***0*.*79***	***0*.*004***	--
		Head Height to shoulder width (SM)	5.24	±	0.53	5.34	±	0.41	0.16	--
		Shoulder height-to-shoulder width (SM)	4.51	±	0.47	4.60	±	0.36	0.15	--
		Height-to-shoulder diameter (3D)	5.12	±	0.47	5.21	±	0.39	0.19	-1.380
		Shoulder height-to-shoulder diameter (3D)	3.99	±	0.39	4.06	±	0.34	0.20	3.851

Clinical pelvimetry showed a higher percentage of women in the CPD group had diagonal conjugate scored as reached, pelvic side walls scored as convergent, sacrospinous ligament scored as not accommodating two fingers, ischial spines scored as prominent, subpubic angle scored as not accommodating two fingers, and intertuberous ligament scored as not accommodating the knuckles of the fist. Kinect skeletal mapping measurements for head, face, neck, shoulder, hip, and right and left leg heights, right femur length, and shoulder-to-head length were significantly smaller in the CPD group compared to the VD group. Kinect 3D camera measurements for height, shoulder height, hip height, head length, hip-to-shoulder length, hip diameter, diameter 1, and head diameter were significantly lower in the CPD group compared to the VD group. Belly Height-to-Hip Diameter (3D), Belly Height-to-Hip Diameter (3D), Torso length-to-Hip height (3D), Height-to-Belly height (3D), and Head height (SM)-to-Belly height (3D) all differed between CPD and VD groups.

### Anthropometry and CPD risk

Of all the individual anthropometric features, height alone showed the greatest predictive power (Table 2). Height alone identified 36% of the CPD cases as *very high risk* and 40% of the vaginal delivery cases as *mild risk* with likelihood ratios of 4.4 and 4.2 for CPD and VD, respectively (Table 3); however, the remaining CPD cases and vaginal deliveries, for women of height between 151–160 cm showed poor likelihood ratios. Therefore, height alone is a poor predictor of CPD risk for women of medium stature.

**Table 2:** Area under the receiver operator characteristic (ROC) curve. The novel risk scores from Eq (1), with Eq (2)_2_ based on MRI and Kinect measurements, outperformed all other parameters and published CPD indices. The MRI- and Kinect-based CPD risk scores were the only scores with an *AUC*_*adj*_ > 0.80, indicating very good predictive capability. The novel risk scores from Eq (1), with Equation (2)_1_ based on anthropometry were the only score with an *AUC*_*adj*_ = 0.79, was the next best model. *AUC* in the range of 0.70–0.80 are generally considered to have fair predictive capabilities and those in the range of 0.60–0.70 are generally considered poor predictors. Note: *AUC* = area under the receiver operator characteristic curve, S.E. = standard error, C.I. = confidence interval, *HC* = maternal head circumference, SM = skeletal mapping, FPI = fetal pelvic index. *p* < 0.05 denotes statistical significance against the null hypothesis that the *AUC* = 0.50.

			*AUC* ± S.E.			(95% C.I.)		*p*
***Anthropometry***								
	Height		0.742	±	0.037	(0.669 -	0.814)	<0.001
	Hip Height		0.722	±	0.036	(0.652 -	0.793)	<0.001
	HC-to-Height		0.708	±	0.038	(0.633 -	0.783)	<0.001
***Clinical Pelvimetry***								
	Pelvic side walls		0.661	±	0.046	(0.571 -	0.712)	0.001
	Ischial spines		0.636	±	0.049	(0.540 -	0.732)	0.004
	Fetal head station		0.626	±	0.043	(0.541 -	0.717)	0.007
***Kinect Measurements***								
	Head height (SM)		0.740	±	0.039	(0.663 -	0.817)	<0.001
	Neck height (SM)		0.738	±	0.039	(0.661 -	0.815)	<0.001
	Face height (SM)		0.736	±	0.040	(0.658 -	0.814)	<0.001
***MRI Measurements***								
	Inlet Area		0.760	±	0.039	(0.683 -	0.838)	<0.001
	Inlet Circumference		0.758	±	0.040	(0.680 -	0.836)	<0.001
	Diagonal Conjugate		0.749	±	0.040	(0.670 -	0.828)	<0.001
***MRI-based CPD Indices***								
	Mengert, 1948^[^^13^^]^		0.760	±	0.039	(0.683 -	0.838)	<0.001
	Friedman & Taylor, 1969 ^[^^17^^]^: inlet		0.730	±	0.039	(0.654 -	0.807)	<0.001
	Morgan et al., 1986 ^[^^19^^]^: FPI		0.616	±	0.047	(0.524 -	0.708)	0.011
	Sporri et al., 2002 ^[^^18^^]^		0.571	±	0.046	(0.480 -	0.662)	0.12
	Abitbol et al., 1991^[^^16^^]^		0.556	±	0.046	(0.465 -	0.647)	0.22
	Friedman & Taylor, 1969 ^[^^17^^]^: mid-pelvis		0.551	±	0.046	(0.460 -	0.642)	0.26
***Novel Risk Scores***		***AUC***_***adj***_	***AUC***_***best***_**± S.E.**			**(95% C.I.)**		***p***
	MRI-based score ^a^	0.825	0.904	±	0.022	(0.860 -	0.947)	<0.001
	Kinects-based score ^a^	0.801	0.871	±	0.032	(0.807 -	0.934)	<0.001
	Anthropometry-based score ^b^	0.793	0.824	±	0.031	(0.764 -	0.884)	<0.001
	Pelvimetry-based score ^b^	0.721	0.732	±	0.042	(0.650 -	0.815)	<0.001

*a* denotes that this score uses Eq (2)_2_

*b* denotes that this score uses Eq (2)_1_.

**Table 3:** 2x2 Contingency table and 4-point risk score contingency table for select indices. Tabulates the percentage of CPD and VD that scored in the range indicated. For example, 68% of the CPD cases had height ≦156.1 cm and 27% of VD had height in the range greater 156.1 cm and less than 160.3 cm. The 2x2 contingency table for the Kinect risk score shows that 84% of the CPD cases and 87% of the VD can be deected with a FNR of 13% and a FPR of 16%. The 4-point scale for the Kinect risk score shows that 78% of the CPD cases were scored as *very high risk* with a FPR ≤10% and 74% of the VD were scored at mild risk with a FNR ≤10%. Note: CPD = cephalopelvic disproportion, VD = vaginal delivery, FPI = fetal pelvic index, *LR*_*CPD*_ = CPD likelihood ratio = *TPR*/*FPR* within the range of risk values of interest, TPR = true positive rate, *FPR* = false positive rate, *LR*_*VD*_ = vaginal delivery likelihood ratio = *TNR*/*FNR* within the range of risk values of interest, *TNR* = true negative rate, and *FNR* = false negative rate. Green shaded areas denote ‘true’ predictions (i.e., *TPR* or *TNR*) and the orange shaded areas denote ‘false’ predictions (i.e., *FPR* or *FNR*).

	2x2 Contingency Table				4-point Risk Score Contingency Table							
	*Predicted CPD*		*Predicted VD*		*Very High*		*High*		*Moderate*		*Mild*	
**FPI**	(≧5.0)		(<5.0)		(≧9.4)		(5.0–9.3)		(4.9 –(-1.8))		(≦-1.9)	
CPD (Actuals)	(57%)		(43%)		(28%)		(29%)		(33%)		(10%)	
VD (Actual)	(43%)		(57%)		(10%)		(34%)		(41%)		(16%)	
* *	*LR*_*CPD*_ *=*	1.32	*LR*_*VD*_ *=*	1.32	*LR*_*CPD*_ *=*	2.84	*LR*_*CPD*_ *=*	0.88	*LR*_*VD*_ *=* 1.24		*LR*_*VD*_ *=*	1.57
**Height**	(≦156.1)		(>156.1)		(≦151.3)		(151.2–156.1)		(156.2–160.2)		(≧160.3)	
CPD (Actuals)	(68%)		(32%)		(36%)		(32%)		(23%)		(9%)	
VD (Actual)	(33%)		(67%)		(8%)		(25%)		(27%)		(40%)	
	*LR*_*CPD*_ *=*	2.05	*LR*_*VD*_ *=*	2.08	*LR*_*CPD*_ *=*	4.37	*LR*_*CPD*_ *=*	1.28	*LR*_*VD*_ *=* 1.19		*LR*_*VD*_ *=*	4.23
**Clinical Pelv.**	(≧0.168)		(<0.168)		(≧0.368)		(0.168–0.367)		(0.132–0.167)		(≦0.131)	
CPD (Actuals)	(72%)		(28%)		(22%)		(50%)		(18%)		(10%)	
VD (Actual)	(44%)		(56%)		(4%)		(40%)		(12%)		(44%)	
	*LR*_*CPD*_ *=*	1.64	*LR*_*VD*_ *=*	2.00	*LR*_*CPD*_ *=*	5.56	*LR*_*CPD*_ *=*	1.24	*LR*_*VD*_ *=*	0.66	*LR*_*VD*_ *=*	4.45
**Mengert**	(≦424)		(>424)		(≦387)		(388–423)		(424–462)		(≧463)	
CPD (Actuals)	(73%)		(27%)		(41%)		(31%)		(18%)		(10%)	
VD (Actual)	(31%)		(69%)		(10%)		(21%)		(32%)		(37%)	
	*LR*_*CPD*_ *=*	2.34	*LR*_*VD*_ *=*	2.52	*LR*_*CPD*_ *=*	4.00	*LR*_*CPD*_ *=*	1.53	*LR*_*VD*_ *=*	1.80	*LR*_*VD*_ *=*	3.81
**Anthro**	(≧0.189)		(<0.189)		(≧0.380)		(0.189–0.379)		(0.097–0.188)		(≦0.096)	
CPD (Actuals)	(75%)		(25%)		(53%)		(23%)		(15%)		(9%)	
VD (Actual)	(29%)		(71%)		(10%)		(19%)		(24%)		(47%)	
	*LR*_*CPD*_ *=*	2.64	*LR*_*VD*_ *=*	2.91	*LR*_*CPD*_ *=*	5.45	*LR*_*CPD*_ *=*	1.20	*LR*_*VD*_ *=*	1.59	*LR*_*VD*_ *=*	5.03
**Kinect**	(≧0.232)		(<0.232)		(≧0.346)		(0.232–0.345)		(0.145–0.231)		(≦0.144)	
CPD (Actuals)	(80%)		(20%)		(57%)		(23%)		(11%)		(9%)	
VD (Actual)	(18%)		(82%)		(10%)		(8%)		(15%)		(67%)	
	*LR*_*CPD*_ *=*	4.46	*LR*_*VD*_ *=*	4.02	*LR*_*CPD*_ *=*	5.95	*LR*_*CPD*_ *=*	2.74	*LR*_*VD*_ *=*	1.35	*LR*_*VD*_ *=*	7.36
**MRI**	(≧0.209)		(<0.209)		(≧0.367)		(0.209–0.366)		(0.121–0.208)		(≦0.120)	
CPD (Actuals)	(80%)		(20%)		(65%)		(14%)		(12%)		(8%)	
VD (Actual)	(20%)		(80%)		(10%)		(10%)		(11%)		(69%)	
	*LR*_*CPD*_ *=*	3.93	*LR*_*VD*_ *=*	3.91	*LR*_*CPD*_ *=*	6.65	*LR*_*CPD*_ *=*	1.37	*LR*_*VD*_ *=*	0.90	*LR*_*VD*_ *=*	8.43

**Fig 4:**
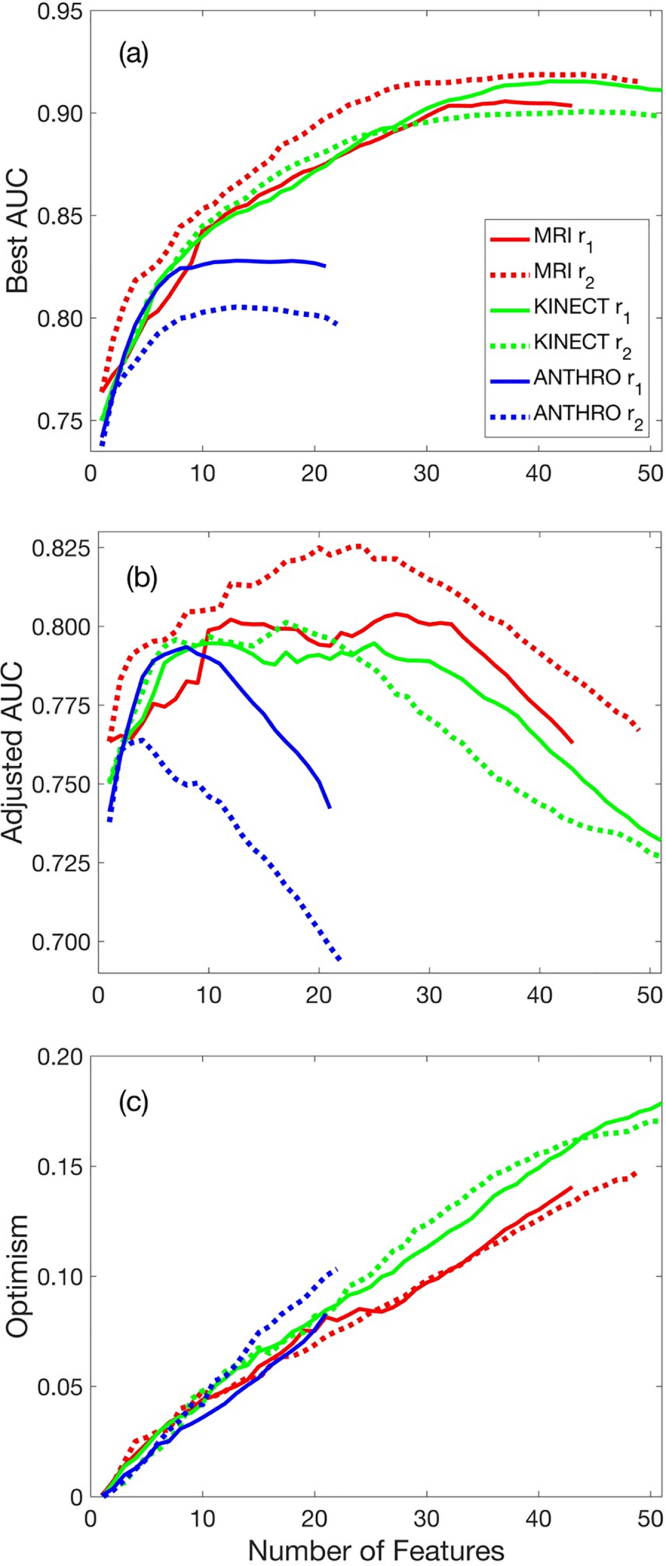
*AUC*_*best*_, *AUC*_*adj*_ and *O* versus number of features (*k*) included in the model. (a) *AUC*_*best*_ increases monotonically with increasing number of features. (b) *AUC*_*adj*_ − *k* curves show that as the number of features included in the model increased, the *AUC*_*adj*_ increased to reach a maximum value, plateaued in some cases, then decreased in models with a high number of features. (c) The optimism, *O*, increased linearly with an increasing number of features.

**Fig 5:**
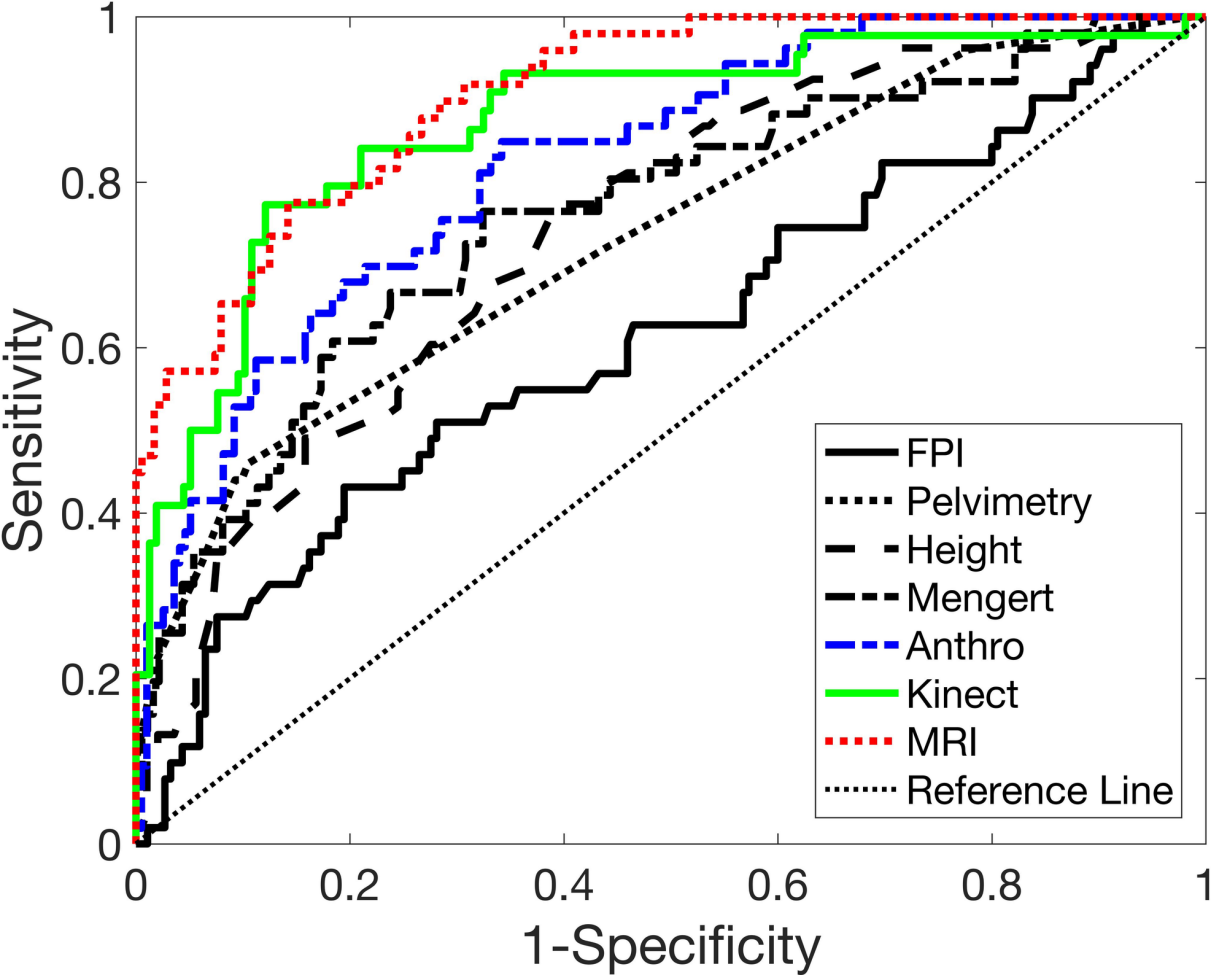
Receiver operator characteristic (ROC) curves. The ROC curves for fetal pelvic index (FPI), clinical pelvimetry, height, the MRI score of Mengert, and the novel anthropometry-, clinical pelvimetry-, Kinect-, and MRI-based risk scores. The area under the ROC curves, shown in Table 2, for MRI-, Kinect-, anthropometry-, clinical pelvimetry-based scores, Mengert, height, and FPI.

The anthropometry-based risk model, following Eq (1) with Eq (2)_1_ yielded a higher *AUC*_*best*_ and *AUC*_*pred*_, compared to Eq (2)_2_ (Fig 4). An 8-parameter risk model yielded the highest *AUC*_*pred*_ = 0.793, with the corresponding *AUC*_*best*_ = 0.824 (Table 2 and Fig 5). Model parameters (*β*_*o*_ and *β*_*i*_) for each model are provided in Table 1. The optimism, *O*, increased linearly with the number of features. This risk model was able to identify 53% of the CPD cases as *very high risk* and 47% of the vaginal delivery cases as *mild risk*, with likelihood ratios of 5.5 and 5.0 for CPD and VD, respectively (Table 3); the remaining CPD cases and vaginal deliveries, for the *high risk* and *moderate risk* range, showed likelihood ratios of 1.2 and 1.6 for CPD and VD, respectively.

### Clinical pelvimetry and CPD risk

Of all the individual features from clinical pelvimetry, pelvic side walls showed the greatest predictive power, followed by ischial spines and fetal head station. A 4-parameter risk model yielded the highest *AUC*_*pred*_ = 0.721, with the corresponding *AUC*_*best*_ = 0.732; model parameters (*β*_*o*_ and *β*_*i*_) for each model are provided in Table 1. This risk model was able to identify only 22% of the CPD cases as *very high risk* and 44% of the vaginal delivery cases as *mild risk*.

### Radiological pelvimetry and CPD risk

The inlet area (*AUC* = 0.760), which is proportional to the metric of Mengert [13], was the best individual or previously published metric based on radiological pelvimetry, followed by inlet circumference and diagonal conjugate. The model of Abitbol *et al*.[16] was a poor predictor of CPD in this subject sample, with *AUC* = 0.556, which was not statistically different from the *AUC* = 0.50, which indicates a random sampling. We found that the model of Friedman and Taylor [17] based on inlet capacity was a fair predictor, but that based on midpelvic capacity was a poor predictor. The index of Sporri *et al*. was also a poor predictor, since mid-pelvic capacity was typically lower than inlet capacity [18]. We found that the *FPI* was also a marginal predictor of CPD, with an *AUC* = 0.616 [19].

The MRI-based risk model, following Eq (1) with Eq (2)_2_ yielded a higher *AUC*_*best*_ and *AUC*_*pred*_, compared to Eq (2)_1_ (Fig 4). A 24-parameter risk model yielded the highest *AUC*_*pred*_ = 0.825, with the corresponding *AUC*_*best*_ = 0.906; model parameters (*β*_*o*_ and *β*_*i*_) for each model are provided in Table 1. The novel MRI risk model yielded the highest *AUC*_*best*_ and *AUC*_*adj*_ of all risk models (Figs 4 and 5) and identified 65% of the CPD cases as *very high risk* and 69% of the vaginal delivery cases *mild risk* with high likelihood ratios of 6.7 and 8.4 for CPD and VD, respectively; the remaining CPD cases and vaginal deliveries, for the *high risk* and *moderate risk* range, showed likelihood ratios of 1.4 and 0.9 for CPD and VD, respectively.

### Kinect 3D camera measurements and CPD risk

Of all the individual Kinect measurements, head height, neck height, and face height from the skeletal map, showed the greatest predictive power; each was on par with anthropometric height. The Kinect-based risk model, following Eq (1) with Eq (2)_1_ yielded a higher *AUC*_*best*_, but Eq (2)_2_ yielded a higher *AUC*_*pred*_ (Fig 4). A 17-parameter risk model yielded the highest *AUC*_*pred*_ = 0.801, with the corresponding *AUC*_*best*_ = 0.871 (Fig 5); model parameters (*β*_*o*_ and *β*_*i*_) for each model are provided in Table 1. The novel Kinect-based risk score identified 57% of the CPD cases as *very high risk* and 67% of the vaginal delivery cases as *mild risk* with high likelihood ratios of 6.0 and 7.4 for CPD and VD, respectively; the remaining CPD cases and vaginal deliveries, for the *high risk* and *moderate risk* range, showed likelihood ratios of 2.7 and 1.4 for CPD and VD, respectively.

### Risk score evaluation and assessment

The two models considered in this study, r_1_ and r_2_, showed differing results across risk modalities. For MRI and Kinect, r_2_ generated a higher *AUC*_*adj*_, compared to r_1_, whereas for the anthropometry risk score, r_1_ was superior to r_2_. For each model, as the number of features included in the model (*k*) increase, the *AUC*_*best*_ also increased asymptotically to a maximum value (Fig 4A). For Kinect, the asymptotic value of *AUC*_*best*_ was higher for r_1_, compared to r_2_, even though r_2_ yielded the higher *AUC*_*adj*_. In contrast, as the number of features included in the model increased, the *AUC*_*adj*_ increased to reach a maximum value, plateaued in some cases, then decreased in models with a high number of features (Fig 4B). Thus, although the *AUC*_*best*_ generally increases monotonically with an increasing number of features, the most predictive models will likely correspond to a model with a moderate number of features. Indeed, for these models, the optimism, *O*, increased linearly with an increasing number of features (Fig 4C). Generally, at lower values of *k*, the slope of the *AUC*_*best*_ − *k* curve (Fig 4A) is greater than the slope of the optimism *O* − *k* curve (Fig 4C), so that the slope of the *AUC*_*adj*_ − *k* (Fig 4B) is positive. In contrast, at higher values of *k*, the slope of the *AUC*_*best*_ − *k* curve is less than the slope of the optimism *O* − *k* curve, so that the slope of the *AUC*_*adj*_ − *k* is negative. The maximum *AUC*_*adj*_ occurs at values of *k* in which the slope of the *AUC*_*best*_ − *k* curve is equal to that of the *O* − *k* curve.

## Discussion

Given that Ethiopia’s density of doctors is 1 doctor for every 40,000 people, a large majority of women who seek care do not see an Obstetrician and Gynecologist during antenatal care and delivery [68]. In most cases, care is provided by a midwife or health extension worker. While clinically trained obstetricians and gynecologists have built intuition, based on training and experience, most of the antenatal care givers in Ethiopia lack the knowledge and expertise to identify patients at risk of obstructed labor due to CPD. The goal of this paper is to develop and validate simple tools to translate a clinician’s intuition to a rural midwife or health worker in Ethiopia, by providing an automatic, safe, easy-to-use, easy-to-interpret, low-cost assessment of risk of CPD.

### CPD risk assessment from anthropometry

Height has long been recognized as a risk indicator for C/S due to CPD [22–24] across multiple nationalities and regions of the world [22, 25–35], including sub-Saharan Africa [24, 36–46]; however, the sensitivity and specificity of height as a predictor of CPD are generally low [69]. The specificity, sensitivity, and likelihood ratios reported herein are consistent with many reports in the literature [24, 27, 31, 38, 40, 44, 46, 52, 69–73]. Other anthropometric features or combinations of features have also been identified as potential risk indicators for CPD, including foot length [25, 35, 42, 45, 47], shoulder diameter [35], lower limb length [42], BMI [32, 48–51], maternal head circumference-to-height ratio [32], and external pelvimetry (e.g., external intercrestal, interspinous, intertrochanteric and intertuberous transverse pelvic diameters, antero-posterior external conjugate or Baudelocque diameter; and transverse and vertical diagonals of the Michaelis sacral rhomboid area) [28, 34, 35, 45, 52]. CPD risk assessment based on anthropometry have shown low sensitivity and specificity [32–35, 45, 52, 55, 56]. For the current study, all height measurements, shoulder width, foot length were lower, and head circumference-to-height ratio and BMI were higher with CPD versus VD. The external pelvic dimension measured in this study were not different across groups. We did not measure the sacral rhomboid dimensions with a tape measure, but did measure these distances from our MRI measurements; the vertical and transverse dimension were lower with CPD. Nevertheless, in the current study, height alone out-performed (i.e., yielded a higher *AUC* value than) foot length, shoulder diameter, BMI, maternal head circumference-to-height ratio, and all external pelvimetry features as a predictor of CPD.

The proposed novel CPD risk score based on anthropometry, showed an *AUC*_*best*_ = 0.824 and *AUC*_*adj*_ = 0.793; the latter was higher than any previously reported risk score that we considered herein, applied to this group of Ethiopian women. Typically, *AUC* in the range of 0.80 and 0.90 are considered good predictors, whereas those between 0.70 and 0.80 are considered only fair predictors. In the current study, the tape measurements were made by nurses trained by the research team and under the direct observation of the research team, to minimize measurement errors, whenever possible. In the field, however, traditional anthropometry is prone to measurement errors and shows poor inter- and intra-user variabilities, which may limit the scale up potential of this approach [54, 74–76]. Examples include the following: (*i*) Improper positioning of the subject; e.g., the subjects’ heels and back are not flush to the length board, the subjects’ head is tilted forward or to the side or not held in the Frankfort plan, the subject’s knees are bent, the subjects’ feet are not flat, the subject is wearing shoes, or the subjects’ posture is not completely upright when measuring height. (*ii*) Improper positioning of the tape measure; e.g., not measuring hip or waist circumference at the widest part of the of the hip or waist or head circumference measurements are not taken at appropriate location on the forehead. (*iii*) Insufficient (or excess) tension in the measuring tape or including clothing under measuring tape. (*iv*) Errors in reading or recording of values from the tape measure. (*v****)*** Broken, poor quality, or inaccurate length boards and tape measures. (*iv*) Inaccurate calculations (e.g., waist-to-hip ratio), (*vi*) Dujardin *et al*. suggests that errors occur due to the readers attraction to round numbers, assignment of some standard sizes, and intentional over-recording of at-risk women [53]. Nevertheless, once validated against a larger sample size, this CPD risk assessment approach, based on anthropometry, could be packaged into an easy-to-use smartphone or computer application to provide a low-cost, easy-to-use tool to assess risk with good predictive capability.

### CPD risk assessment from clinical pelvimetry

Clinical pelvimetry assesses the size of the pelvic cavity by means of the systematic vaginal palpation of specific bony landmarks in the pelvis and an estimation of the distances between them. The proposed novel CPD risk score based on pelvimetry, based on Eq (1), using Eq (2)_1_ with the parameters specified in Table 1, yielded an *AUC* below that of height alone. One limitation to scaling this approach is that this technique requires highly skilled personnel [57, 58, 77]. Given that Ethiopia’s density of doctors (0.025/1000 people) and nurse/midwifery personnel (0.252/1000 people) are amongst the lowest in the world [68], the number of qualified personnel that could be trained in this technique in Ethiopia is too low to effectively scale. Further, given that this technique is subjective and shows only fair predictive capability, scaling this technique may not be a useful investment of resources [57, 58, 77].

### CPD risk assessment from radiological pelvimetry

Use of X-ray, and later ultrasound, MRI, and CT to measure fetal and pelvic dimensions, and assess risk of adverse pregnancy outcomes including CPD, has been an active area of research for nearly a century [13–20]. When applied to the Ethiopian women in our cohort, aside from the risk scores from Mengert [13] and Friedman and Taylor [17], other published radiological pelvimetry-based risk scores proved to be poor predictors of CPD; however, our novel CPD risk score based on MRI measurements showed the highest *AUC*_*adj*_. MRI and CT are cost prohibitive in low-resource settings and a majority of mothers in the developing world do not have access to MRI or CT. X-ray machines are more readily available, but radiation exposure to the fetus poses an unwanted health risk and may represent a significant barrier to widespread scale-up in the developing world. Assessment of fetal size can be done via ultrasound imaging; however, ultrasound technology and expertise are also limited in most low-resource settings. Further, the accuracy of radiological pelvimetry at predicting CPD-related obstructive labor is controversial and lacks testing via rigorous randomized trials [14, 21]. In the current study, since we conducted MRI to quantify of the maternal pelvic dimensions, we also assessed fetal dimensions using MRI. Ultrasound fetal measurements were not routinely collected on study participants during their normal antenatal care; thus, to collect these data would require an additional exam, which we felt was unwarranted.

### CPD risk assessment from kinect 3D camera

We developed a low cost, safe, easy-to-use, portable platform to assess risk of obstructed labor due to CPD, by employing a Kinect 3D camera. The automated Kinect-based CPD risk score showed very good predictive capabilities and performed better than all previously published metrics. Automated measurements, through the use of 3D camera technology offer the potential to significantly reduce measurement errors and the inherent inter- and intra-user variabilities in measurement, compared to tape measurements, thereby providing a more reliable assessment. Further, whereas length board and tape measurements are limited to ‘linear’ measurements of exterior landmarks, camera technology offers the possibility of producing new measurements that cannot be measured with a tape measure. Examples include the following: (*i*) Total volume of a certain region (e.g., the arm, leg, or fundus) can be accurately and automatically measured from 3D models of a subject. (*ii*) Curvatures (e.g., curvature of the hips or fundus) can be measured from 3D images. (*iii*) The skeletal mapping feature of the Kinect sensor and software offer the ability to obtain ‘internal’ dimensions; e.g., novel algorithms, developed by Microsoft, conduct real-time analysis to accurately estimate 25 joint locations (e.g., hip, shoulder, and knee joints). For two women with the same pelvic dimensions, one woman at a healthy weight and the other overweight, the hip and waist circumference of these two women may be very different, but the hip joint location, determine via skeletal mapping may be similar. (*iv*) 3D models and skeletal maps can also be helpful in measuring accurate heights, even if the subject is not positioned properly. For example, if the participants knees are bent and/or their back is not perfectly vertical, the sum of the distance, in 3-dimension, from the ankle joint to the knee joint, the knee joint to the hip, the hip to the shoulder, and the shoulder to the top of the head eliminate the errors due to bending of the subject out of the measurement plane. (*v*) Some measurements have been shown to be useful indicators of CPD, that require calipers (anthropometer) to measure; e.g., external conjugate and inter-trochanteric diameter[35]. These measurements can be accurately and automatically measured from 3D models of a subject, without the need for an anthropometer.

### Limitations of this study

The current study has several important limitations that should be mentioned. (*i*) Given the small sample size of this feasibility study, the proposed risk models require additional refinement, based on a larger sample size and cross-section of the population, and a broader, blinded, prospective, validation studies before this method can be applied clinically. (*ii*) We evaluated two risk models (Eq 1, with r_1_ and r_2_); however, there are numerous other options that can be taken from machine learning and data science that should be evaluated in future work. (*iii*) This study relied on recruitment of women with previous obstetric history of CPD, some of whom did not try labor. Thus, it is possible that some of the participants that underwent elective C/S could have successfully delivered vaginally in the current pregnancy. (*iv*) With regard to the clinical pelvimetry, one limitation is that the examiners were not blinded to the previous obstetric history. (*v*) We have not assessed the participant and caregivers views, perception, and acceptability of the Kinect 3D camera. Although the Kinect camera is safe and has no unacceptable exposure limit, participant and caregivers perceived risk of using this tool may be an important barrier that should be quantified and addressed in future studies.

(*vi*) We did not include some parameters as variables in the risk model, because these data are potentially biased across our experimental groups. Labor is a multivariate process, influenced by multiple factors that were not included in the current risk model; factors such as maternal age, parity, obstetric history, gestational age, paternal factors, amongst others. The sample population used in this study is not a representative cross section of all pregnant mothers from the TASR hospital catchment area. For example, the high-risk group (Group 1) was older than our primigravida group (Group 3). This is not necessarily associated with CPD; rather, this is (at least partly due) to our experimental design, since any group of multigravida women will, on average, be older than a group of primigravida women from the same population.

(*vii*) All measurements were taken at >36 weeks of gestation; there is a need to develop tools that accurately predict CPD over the 1^st^, 2^nd^, and 3^rd^ trimester. We believe that many of the key features in our preliminary risk score will not change significantly throughout gestation; e.g., maternal height, maternal shoulder diameter, or maternal head circumference. Therefore, a risk score that is accurate, based on measurements made at 36–42 weeks of gestation, may be modified to be accurate based on measurements made at any gestational age. Validating this risk assessment tool across a wide range of gestational ages will expand the tools utility; e.g., healthcare providers could assess risk at multiple gestational ages, which could help identify risk earlier in pregnancy and affirm risk assessment after multiple measurements. Given that it may require hours or days of travel for mothers in the rural developing world to reach facilities capable of performing a C/S, early and accurate diagnosis of risk of CPD may be crucial for mothers to plan for labor and delivery. Further, given that a majority of women in Ethiopia have 3 or fewer antenatal care visits [7], expanding the validated gestational age range across which this tool is valid may increase the number of pregnant women who access the tool.

(*viii*) The Kinect skeletal mapping joint locations are not an accurate representation of the actual anatomical joint locations. Thus, data on the actual joint locations should be used with caution. That said, we expect that the skeletal mapping outputs are repeatable measurements, despite not accurately representing the actual anatomical locations, that may be important features used to assess risk of CPD. (*ix*) The scan-acquisition method employed in this study captured 3D point clouds from four orthogonal views (anterior, posterior, left and right lateral). In many cases, this method limited our ability to stitch these four point-clouds together into an accurate 3D model due to lack of common landmarks across views. In the future, an improved acquisition protocol, with a larger number of views is recommended [78].

### Opportunities and limitations to scale-up

Our long-term goal is to develop novel strategies to improve the diagnosis of risk of CPD in areas where emergency C/S is not readily available during labor. Despite a century of work towards developing such a tool, no tool to assess risk of CPD is adopted as standard clinical practice. This is likely because of two distinct reasons: (*i*) given the nearly universal access to emergency C/S in the developed world, the utility of such a tool in the developed world has diminished; and, (*ii*) previous risk scores have shown only fair predicative capabilities. Nevertheless, the pressing need remains for accurate and timely diagnosis of CPD in the rural developing world, where access to C/S may require significant travel for mothers. With the rapid advancement both in the areas of low-cost 3D camera technology and in data science, there is a unique opportunity to develop such a low cost, usable tool.

Although the primary goal of this paper was to demonstrate the feasibility of using a Kinect 3D camera to assess risk of CPD, and important secondary outcome is the development of a tape measurement-based tool, which is also a novel contribution of this work and may be an important part of the potential scale up strategy. Although research has shown that some anthropometric features correlate to risk of CPD, such measurements are not currently used to assess risk of CPD. Indeed, in Ethiopia, CPD typically goes undiagnosed. Given its ultralow cost and ease of use, the novel tape measurement-based risk score may provide and important initial screening to ‘triage’ low risk subjects and refer potentially high-risk subjects for further screening with a 3D camera.

One potential barrier to wide-spread uptake and participant acceptability is the need for participants to disrobe during the Kinect scan. For the purpose of this feasibility study, subjects’ were asked to disrobe, with the exception of tight-fitting undergarments, as clothing would interfere with the generation of representative 3D measurements that may be important in predicting CPD; e.g., the fundal geometry, waist circumference, hip circumference, etc. Thus, for this feasibility study, we sought to identify all of these potentially important parameters by scanning disrobed subjects. That said, one may imagine a risk score that is based only on measurements that could be collected while the subject is clothed; e.g., heights, shoulder dimensions, and most skeletal mapping outputs. Indeed, the Kinect scanner is designed to perform these analyses on clothed subjects. Such analysis was not included in the current study since additional measurement error may be introduced when data is collected from clothed participants but should be a focus of future studies.

### Conclusions

A novel application of the Kinect 3D camera to assess risk of CPD is presented, which performed on par with the novel MRI-based score and outperformed all previously reported risk scores applied to this group of Ethiopian women. This work demonstrates the feasibility of a safe, low-cost, easy-to-use 3D camera technology to assess risk of CPD. This work demonstrates the need for further broader blinded, prospective studies to refine and validate the proposed CPD risk scores, which are required before this method can be applied clinically.

## Supporting information

S1 Table Data collection form used for ensuring the participants meet the eligibility criterion (Section 1) and recording anthropometric measurements (Section 2), clinical pelvimetry (Section 3), radiological pelvimetry (Section 4), and pregnancy outcomes (Section 5). The columns in S2 Table correspond to the rows in this data collection form. This Table is provided in the supplemental file named “S1_Table.docx”.
(DOCX)

S2 Table Raw data for all participants for eligibility criteria, anthropometry, clinical pelvimetry, radiological pelvimetry, and pregnancy outcomes. The columns in S1 Table correspond to the rows in this data collection form. This Table is provided in the supplemental file named “S2_Table.csv”.
(CSV)

## Abbreviations

3D: Three-dimensional
A: Cross-sectional area
APD: Anterior-posterior diameter
AUC: Area under the receiver-operator curve
BMI: Body mass index
BPD: Fetal head biparietal diameter
C: Circumference
CPD: Cephalopelvic disproportion
C/S: Caesarean section
CT: Computed tomography
FPI: Fetal-pelvic index
MRI: Magnetic resonance imaging
OFD: Occipitofrontal diameter
OMD: Occipitomental diameter
PSD: Posterior sagittal diameter
ROC: Receiver-operator curve
SOBD: Suboccipitobregmatic diameter
TD: Transverse diameter
V: Volume/pelvic capacity
