# Supplementary material for: A safe, low-cost, easy-to-use 3D camera platform to assess risk of obstructed labor due to cephalopelvic disproportion
Source: PLoS One. 2018 Sep 14;13(9):e0203865. doi: 10.1371/journal.pone.0203865 (PMC6138392; doi:10.1371/journal.pone.0203865)
Supplement: S1 Table — The columns in S2 Table correspond to the rows in this data collection form. This Table is provided in the supplemental file named “S1_Table.docx”. (DOCX) [file pone.0203865.s001.docx]

###### Section 1: Eligibility Criterion (during antenatal visit at ~37 weeks)

| Q No. | Questions and Filters | Codes | | Response | | | |
| --- | --- | --- | --- | --- | --- | --- | --- |
| 101 | Participant Identification Number | ##### | | BL-00 _____ _____ _____ | | | |
| 102 | Participants Initials | XXX | | _____ _____ _____ | | | |
| 103 | Participants Hospital Number | ###### | | _____ _____ _____ _____ _____ _____ | | | |
| 104 | Initials of recruiting nurse | XXX | | _____ _____ _____ | | | |
| 105 | Date of recruitment & filling out this form.  USE ETHIOPIAN CALENDAR | Date ____ ____ / ____ ____ / ____ ____ ____ ____  (day) (month) (year) | | | | | |
| 106 | How old was the participant on their last birthday?  PROBE USING HISTORICAL EVENTS TO APPROXIMATE AGE, IF UNKNOWN | Age (in years)...… ## | | | | | _____ _____ |
| 107 | Is the subject age between 18 and 40 yrs? | Yes........... 1  No............ 2>>**STOP! Exclusion Criteria** | | | | | _____ |
| 108 | Does this participant plan for trial of labor? | Yes........... 1  No............ 2 | | | | | ____ |
| 109 | What is the gestational age of the participant? | Weeks completed + days…##+# | | | | ____ ____ + ____ | |
| 110 | How was the gestational age determined? | Recalled days from last menstruation... 1  Ultrasound......................................................... 2  Other.................................................................... 3 | | | | | ____ |
| 111 | Is this a singleton pregnancy? | Yes........... 1  No............ 2>>**STOP! Exclusion Criteria** | | | | | ____ |
| 112 | Check the fetal presentation. | Vertex.... 1  Other...... 2>>**STOP! Exclusion Criteria** | | | | | ____ |
| 113 | Now I would like to ask about all the births you have had during your life prior to this pregnancy. How many children (to whom you have given birth) are alive? | Children……….................… #  If more than 8.................... 9  If none, enter……………… 0 | | | _____ | | |
| 114 | Have you ever given birth to a child who was born alive but later died? *IF NO, PROBE:* “Any baby who cried and showed signs of life but did not survive?”  How many children have died? | | Children………...........… #  If more than 8.............. 9  If none, enter……….… 0 | | _____ | | |
| 115 | SUM ANSWERS TO 113 AND 114 AND ENTER TOTAL. | Total births……. #  If more than 8... 9  If none, enter…. 0**>> 121 (Group 3)** | | | _____ | | |
| 116 | How were your previous children delivered?  INDICATE FOR EACH CHILD STARTING WITH THE OLDEST CHILD (Child 1) | Vaginally or Instrumental………1  Cesarean Section……………………2  IF ANSWER IS 2 FOR ANY CHILD, PROCEED TO NEXT QUESTION.  IF NO CHILDREN WERE DELIVERED BY CESAREAN SECTION **>> 119 (Group 2)** | | | Child 1: _____  Child 2: _____  Child 3: _____  Child 4: _____  Child 5: _____  Child 6: _____ | | |

###### Section 1: Eligibility Criterion (during antenatal visit at ~37 weeks)

| Q No. | Questions and Filters | Codes | Response |  |
| --- | --- | --- | --- | --- |
| 117 | Were any previous C-sections due to prolonged labor due to CPD? | Yes........... 1  No............ 2 >>**STOP! Exclusion Criteria** | _____ |  |
| 118 | If yes (Q122), how many hours was the participant in labor prior to the CPD related C-section? | Hours......... ### | _____ _____ |  |
| 119 | How much did your youngest child weigh at birth? RECORD WEIGHT IN KG FROM HEALTH / HOSPITAL CARD, IF AVAILABLE | Weight…….. #.# kg | ____ **.** ____ | |
| 120 | Was weight recorded from health/hospital card or from mothers recall? | From health/hospital card..... 1  From recall.................................... 2 | ____ | |
| 121 | How many months pregnant were you when you first received antenatal care for this pregnancy? | Months………... ##  Don’t Know…...98 | ____ ____ | |
| 122 | How many times did you receive antenatal care during this pregnancy prior to this visit? | Number of Times…. ##  Don’t Know………….. 98 | ____ ____ | |
| 123 | To which experimental group is this participant assigned? | Previous C-section due to CPD.... 1  Previous vaginal delivery  (no previous C-sections)........... 2  First pregnancy.................................. 3 | ____ | |
| 124 | If no exclusion criteria are met, schedule with the participant a date and time to return for Clinical Pelvimetry, Anthro-pometry, Kinects Scanning, and MRI Scanning.  IF PARTICIPANT IS NOT WILLING OR ABLE TO RETURN FOR THE 38-WEEK VISIT  >>**STOP! Exclusion Criteria** | DATE AND TIME 38-WEEK VISIT  ____ ____ / ____ ____ - ___ ___ : ___ ___ AM / PM  (day) (month) (time) (circle)  USE ETHIOPIAN CALENDAR & TIME | | |

**Section 2: Anthropometric Measurements (Collected at 38 Weeks)**

| Q No. | Questions and Filters | Codes | | | | | Response |
| --- | --- | --- | --- | --- | --- | --- | --- |
| Participant number & initials | | BL-00### / XXX | | BL-00 _____ _____ _____ / _____ _____ _____ | | | |
| 201 | Initials of nurse collecting anthropometric measurements. | | | | XXX | | _____ _____ _____ |
| 202 | Date of recruitment & filling out this form.  USE ETHIOPIAN CALENDAR | | Date ____ ____ / ____ ____ / ____ ____ ____ ____  (day) (month) (year) | | | | |
| 203 | Height | Height (in cm)……………................ ###.# | | | | | _____ _____ _____ . _____ |
| 204 | Weight | Weight (in kg)……..…….................. ##.# | | | | | _____ _____ . _____ |
| 205 | Fundal height | Height (in cm)……........….............……##.# | | | | | _____ _____ . _____ |
| 206 | Blood pressure | Systolic/diastolic (in  mmHg)......................... ###/### | | | | _____ _____ _____ / _____ _____ _____  SYSTOLIC DIASTOLIC | |
| 207 | Head Circumference | Circumference (in cm)………….... ##.# | | | | | _____ _____ . _____ |
| 208 | Bisacromial (shoulder) width | Width (in cm)…………...................... ##.# | | | | | _____ _____ . _____ |
| 209 | Shoulder height | Height (in cm)……......................…... ###.# | | | | | _____ _____ _____ . _____ |
| 210 | Waist circumference | Circumference (in cm)………........ ###.# | | | | | _____ _____ _____ . _____ |
| 211 | Waist height | Height (in cm)………......................... ###.# | | | | | _____ _____ _____ . _____ |
| 212 | Intertrochanteric (hip) circumference | Circumference (in cm)……........... ###.# | | | | | _____ _____ _____ . _____ |
| 213 | Intertrochanteric height | Height (in cm)……………................. ###.# | | | | | _____ _____ _____ . _____ |
| 214 | Foot Length | Length (in cm)……………................ ##.# | | | | | _____ _____ . _____ |
| 215 | External conjugate | Diameter (in cm)…………..............…..##.# | | | | | _____ _____ . _____ |
| 216 | COLLECT KINECTS SCANS. Check confirming the collection and saving of the Kinects Scans on the external hard drive.  ☐ File name = (Participant#_Participant initials) | | | | | | |

**Section 3: Clinical Pelvimetry from Physical Exam (Collected at 38 Weeks)**

| Q No. | Questions and Filters | Codes | | | Response |
| --- | --- | --- | --- | --- | --- |
| Participant number & initials | | BL-00### / XXX | | BL-00 _____ _____ _____ / _____ _____ _____ | |
| 301 | Initials of Obstetrician performing physical exam for clinical pelvimetry. | | XXX | | _____ _____ _____ |
| 302 | Date of recruitment & filling out this form.  USE ETHIOPIAN CALENDAR | | Date ____ ____ / ____ ____ / ____ ____ ____ ____  (day) (month) (year) | | |
| 303 | Diagonal conjugate. | | Reached.......... 1  Unreached..... 2 | | _____ |
| 304 | Pelvic side walls. | | Divergent….....… 1  Straight................ 2  Convergent........ 3 | | _____ |
| 305 | Sacrospinus ligament accommodates more tham 2 fingers? | | Yes…….. 1  No........... 2 | | _____ |
| 306 | Are Ischial spines prominent? | | Yes…….. 1  No........... 2 | | _____ |
| 307 | Subpubic angle accommodates more than 2 fingers? | | Yes…….. 1  No........... 2 | | _____ |
| 308 | Intertuberous ligament accommodates the fist? | | Yes…….. 1  No........... 2 | | _____ |
| 309 | Fetal head station | | Score........... -3, -2, -1, 0, 1, 2, 3 | | _____ |

**Section 4: Radiological Pelvimetry from MRI**

| Q No. | Questions and Filters | | Codes | | | | Response | |
| --- | --- | --- | --- | --- | --- | --- | --- | --- |
| Participants Hospital Number | | ###### | | | | _____ _____ _____ _____ _____ _____ | | |
| Participant MR Number | | ###### | | | | _____ _____ _____ _____ _____ _____ | | |
| Participant number & initials | | BL-00### / XXX | | | | BL-00 _____ _____ _____ / _____ _____ _____ | | |
| 401 | Initials of Radiologist assessing MRI scans. | | | | XXX | | _____ _____ _____ | |
| 402 | Date of performing MRI.  USE ETHIOPIAN CALENDAR | | | | Date ____ ____ / ____ ____ / ____ ____ ____ ____  (day) (month) (year) | | | |
| 403 | Pelvic inlet: Anterior-posterior diameter (a.k.a., Obstetric Conjugate) | | | | Centimeters……##.# | | | _____ _____ . _____ |
| 404 | Diagonal Conjugate | | | | Centimeters……##.# | | | _____ _____ . _____ |
| 405 | Pelvic Inlet: Transverse (axial) diameter. | | | | Centimeters……##.# | | | _____ _____ . _____ |
| 406 | Pelvic Inlet: Oblique diameters. | | | | Centimeters……##.# | | | _____ _____ . _____ (right)  _____ _____ . _____ (left) |
| 407 | Pelvic Inlet Area | | | | cm^2^….................…##.# | | | _____ _____ _____ . _____ |
| 407 | Mid pelvis: Anterior-posterior diameter | | | | Centimeters……##.# | | | _____ _____ . _____ |
| 408 | Mid pelvis: Transverse diameter  (a.k.a. interspinous diameter) | | | | Centimeters……##.# | | | _____ _____ . _____ |
| 409 | Pelvic outlet: Anterior-posterior diameter | | | | Centimeters……##.# | | | _____ _____ . _____ |
| 410 | Pelvic outlet: Transvers diameter  (a.k.a. Intertuberous) | | | | Centimeters……##.# | | | _____ _____ . _____ |
| 411 | Pelvic outlet: Posterior sagittal diameter | | | | Centimeters……##.# | | | _____ _____ . _____ |
| 413 | Sacral rhomboid dimensions | | | Vertical Diagonal (in cm)..........…##.#  Transverse Diagonal (in cm)….. ##.# | | | | _____ _____ . _____  _____ _____ . _____ |
| 414 | Distance between medial femoral heads. | | | | Centimeters……##.# | | | _____ _____ . _____ |
| 415 | Intertrochanteric distance | | | | Centimeters……##.# | | | _____ _____ . _____ |
| 416 | Fetal head: Fetal head area | | | | cm^2^................……###.# | | | _____ _____ _____ . _____ |
| 417 | Fetal head: Bipariental Diameter | | | | Centimeters……##.# | | | _____ _____ . _____ |
| 418 | Fetal head: Occipitofrontal diameter | | | | Centimeters……##.# | | | _____ _____ . _____ |
| 419 | Fetal head: Occipitomental diameter | | | | Centimeters……##.# | | | _____ _____ . _____ |
| 420 | Fetal head: Suboccipitobregnatic diameter | | | | Centimeters……##.# | | | _____ _____ . _____ |
| 421 | Fetal abdomen: Anterior-posterior diameter | | | | Centimeters……##.# | | | _____ _____ . _____ |
| 422 | Fetal abdomen: Transverse diameter | | | | Centimeters……##.# | | | _____ _____ . _____ |

**Section 5: Pregnancy Outcome (Collected after delivery from hospital card)**

| Q No. | Questions and Filters | Codes | | Response |
| --- | --- | --- | --- | --- |
| Participant number & initials | | BL-00### / XXX | BL-00 _____ _____ _____ / _____ _____ _____ | |
| Participants Hospital Number | | ###### | _____ _____ _____ _____ _____ _____ | |
| 501 | Initials of nurse recording pregnancy outcomes. | XXX | | _____ _____ _____ |
| 502 | Did the baby die during labor, delivery or within 7-days after labor? | Yes............... 1  No................ 2 >> 505 | | _____ |
| 503 | What was the cause of death of the baby? | Trauma from Obstructed Labor……. 1  Small for date/pre-term………………. 2  Asphyxia……………………………………... 3  Jaundice....................................................... 4  Infection………..….………………………… 5  Sudden Infant Death Syndrome……. 6  Other…………………………………………... 8  __________________________  (Specify Other)  Unknown…………………………………….. 9 | | _____ |
| 504 | Did the mother die during labor, delivery, or within 7-days after labor and delivery? | Yes............... 1  No................ 2 >> 507 | | _____ |
| 505 | What was the cause of death of the mother? | Obstructed Labor………………………… 1  Hemorrhage / bleeding.……………….. 2  Preeclampsia/eclampsia...................... 3  Other…………………………………………... 8  __________________________  (Specify Other)  Unknown…….………………………………. 9 | | _____ |
| 506 | What is the baby’s birthdate?  Month …………….… 01 – 13  Day………………….…01 – 31  Year………………….. #### | Birthdate ____ ____ / ____ ____ / ____ ____ ____ ____  (day) (month) (year)  **USE ETHIOPIAN CALENDER** | | |
| 507 | How was the baby delivered? | Vaginally or Instrumental………1 >> 511  Caesarean Section……………………2 | | _____ |
| 508 | Why was a Caesarean section performed? | Arrested labor/dystocia........... 1  Abnormal presentation............. 2 >> 511  Fetal distress/cord prolapse..... 3 >> 511  Placental problems..................... 4 >> 511  Failed induction........................... 5 >> 511  Elective, fear of CPD................... 6  Elective, fear of childbirth....... 7 >> 511  Other................................................. 8 >> 511 | | _____ |
| 509 | How many hours was the participant in labor prior to C-section? | Hours......... ### | | _____ _____ _____ |

**Section 5: Pregnancy Outcome (con’t)**

| Q No. | Questions and Filters | Codes | Response |
| --- | --- | --- | --- |
| 510 | What was the baby’s weight **at birth**? | Weight (in kg)…………… #.## | ____ . ____ ____ |
| 511 | What was the baby’s length **at birth**? | Length (in cm)…………… ##.# | ____ ____ . ____ |
| 512 | What was the baby’s head circumference **at birth**? | Circumference (in cm)……##.# | ____ ____ . ____ |
| 513 | What is the baby’s gender? | Male…….............. 1  Female................ 2 | ____ |
| 514 | What was the Apgar score? | Apgar.................. 0 - 10 | ____ ____ 1^st^ minute  ____ ____ 5^th^ minute |
| 515 | Were there any other complications experienced by the baby or mother during labor, delivery, or 7-days after delivery? | Yes............... 1 >> Specify below  No................ 2 | ____ |
|  | Specify other complications or notes: | | |

## 
